# Bifunctional phase-transfer catalysis in the asymmetric synthesis of biologically active isoindolinones

**DOI:** 10.3762/bjoc.11.279

**Published:** 2015-12-15

**Authors:** Antonia Di Mola, Maximilian Tiffner, Francesco Scorzelli, Laura Palombi, Rosanna Filosa, Paolo De Caprariis, Mario Waser, Antonio Massa

**Affiliations:** 1 Dipartimento di Chimica e Biologia "A. Zambelli", Università di Salerno, Via Giovanni Paolo II, 132, 84084-Fisciano, SA, Italy; 2 Dipartimento di Farmacia, Università di Salerno, Via Giovanni Paolo II, 132, 84084-Fisciano, SA, Italy; 3 Institute of Organic Chemistry, Johannes Kepler University Linz Altenbergerstraße 69, 4040 Linz, Austria; 4 Dipartimento di Medicina Sperimentale, Università di Napoli, Napoli, Italy

**Keywords:** asymmetric synthesis, Belliotti (*S*)-PD 172938, heterocycles, isoindolinones cascade reactions, phase transfer catalysts

## Abstract

New bifunctional chiral ammonium salts were investigated in an asymmetric cascade synthesis of a key building block for a variety of biologically relevant isoindolinones. With this chiral compound in hand, the development of further transformations allowed for the synthesis of diverse derivatives of high pharmaceutical value, such as the Belliotti (*S*)-PD172938 and arylated analogues with hypnotic sedative activity, obtained in good overall total yield (50%) and high enantiomeric purity (95% ee). The synthetic routes developed herein are particularly convenient in comparison with the current methods available in literature and are particularly promising for large scale applications.

## Introduction

**Figure 1:**
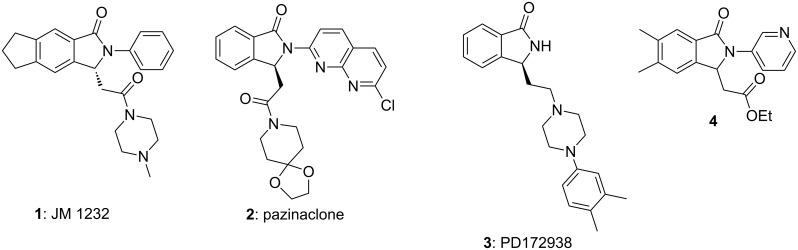
Some chiral, bioactive isoindolinones.

Among the nitrogen heterocycles, the isoindolinone ring system is a favored scaffold, owing to the wide range of applications and pharmacological properties [1–3]. A relevant structural aspect is the presence of a substituent at the C3 position of the ring. A higher biological activity of the enantioenriched compounds with respect to racemic mixtures has been demonstrated for several 3-substituted isoindolinones, some of which are shown in Figure 1 [4–8]. For example, the enantioenriched isoindolinones **1** and **2** are benzodiazepine-receptor agonists for the treatment of anxiety [4–7]. Compound (*S*)-**3**, developed by Belliotti et al. and known as PD172938, is a potent dopamine D_4_ ligand [8] while hypnotic/sedative activity has been investigated only for *rac*-**4** [6–7]. In the last years, the development of the efficient, catalytic, asymmetric synthesis of 3-substituted isoindolinones became a research field of great interest among organic and medicinal chemists [9–23]. Nevertheless, the enantioenriched compounds **1**–**3** and their analogues have as of yet only been accessible via resolution approaches in less satisfactory overall yields (usually <10%). This is accompanied by the need of stoichiometric amounts of valuable chiral resolving agents, illustrating the need for more convenient strategies for large scale applications [4–8].

Recently, cascade reactions have received increasing attention due to the possibility of construction of complex scaffolds in operationally simple, one-pot procedures, starting from readily available materials [24–26]. In this context, our group recently introduced an interesting asymmetric organo-cascade reaction of 2-cyanobenzaldehyde (**5**) with malonate **6**. This resulted in enantioenriched 2-(1-oxoisoindolin-3-yl)malonate **7** which is supposed to serve as a potentially useful building block for further elaborations en route to the targets of medicinal interest (Figure 1). A high yield (usually >95%) and a maximum level of enantioselectivity of 74% ee were obtained but only in the presence of large amounts of cinchona alkaloid-based thiourea-containing organocatalysts (15 mol %) and after an unacceptably long reaction time (72 h) [21–22]. Readily available chiral ammonium salts (e.g., cinchona alkaloid-based or commercially available Maruoka catalysts) were also investigated, but the enantioselectivity was lower, reaching a maximum of 46% ee [23]. Gratifyingly, a very efficient heterochiral crystallization (these isoindolinones crystallize as racemic mixtures) allowed the isolation of the product in high enantiomeric excesses (>90% ee) and good overall yield, making the entire process attractive from a synthetic point of view [21,23]. However, several issues remain to be addressed when targeting the use of such a catalytic strategy to access the target compounds **1**–**4** in an efficient manner.

**Scheme 1:**
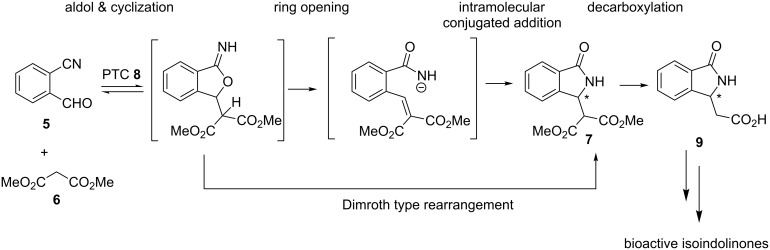
This work: 1) *trans*-1,2-cyclohexane diamine-based bifunctional ammonium salts **8** in the asymmetric synthesis of **7**; 2) transformation of **7** into **9**; 3) asymmetric synthesis of bioactive isoindolinones.

The availability of efficient catalytic processes is a fundamental requirement for the development of scalable synthetic routes of bioactive compounds. Thus, in the present work, we firstly reconsidered this cascade reaction, investigating novel *trans*-1,2-cyclohexane diamine-based bifunctional ammonium salts **8**. These catalysts were recently introduced by our groups in a variety of different reactions [27–29], as exemplified by a related aldol-initiated cascade reaction of glycine Schiff base with 2-cyanobenzaldehydes [29]. Then we decided to address the asymmetric synthesis of bioactive isoindolinones, identifying the chiral isoindolinone **9** as a very useful, key building block, as shown in Scheme 1.

## Results and Discussion

### Asymmetric cascade reaction of 2-cyanobenzaldehyde under phase-transfer conditions

As already discussed [21–23], the bifunctional nature of organocatalysts plays an important role in this reaction to attain satisfactory levels of enantioselectivity. The presence of additional hydrogen donors such as urea groups positively affected the enantioselectivity both in the presence of chiral base organo-catalysts and when using quaternary ammonium salts. In the latter case, however, only 46% ee was obtained using the bifunctional urea-containing ammonium salt catalyst introduced by Dixon's group [30]. Readily available commonly used quaternary ammonium salt catalysts gave racemic mixtures only, although high reactivity was generally observed [23].

Taking advantage of the high flexibility and efficiency of the developed synthetic route to **8** [27,29], a library of more than 20 structurally diversified chiral ammonium salts was tested in the model cascade reaction of 2-cyanobenzaldehyde with dimethyl malonate at room temperature (Table 1). We first identified the combination of DCM and solid K_2_CO_3_ as the best-suited solvent–base system for this reaction, however etheral or aromatic solvents or aqueous (alternative) bases generally gave significantly lower selectivities (this was carefully double-checked once the most active catalyst was identified). High yields were usually observed (>90%) when running the reaction for one night using 5 mol % of the catalyst. Good levels of enantioselectivity (up to 70% ee) were obtained when strong electron-withdrawing groups are present on both the urea and ammonium sides of the catalyst (Table 1, entries 16, 17, and 21). Electron-neutral or bulky aryl groups on the ammonium side, as well as the introduction of a naphthyl group, did not result in any improvement of the enantioselectivity. Also, the presence of aliphatic groups on the urea side did not allow us to improve the catalyst performance and a similar selectivity was obtained upon incorporation of more electron-rich aryl groups R^1^. The initial tests also showed that ureas are more selective than thioureas (Table 1, entry 12). Thus further optimization was carried out with ureas only, finally identifying the catalyst **8a** (R^1^ = 4-NO_2_-C_6_H_4_, R^2^ = 3,5-(CF_3_)_2_-C_6_H_3_) as the most promising one (Table 1, entry 17). It should also be noted that control experiments with catalyst derivatives containing only either a quaternary ammonium or a urea group proved the need for both of these functionalities to obtain satisfactory levels of enantioselectivity.

**Table 1:** Catalyst screening in asymmetric cascade reactions of 2-cyanobenzaldehyde.

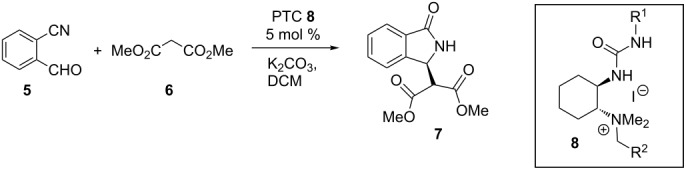				

Entry	R^1^	R^2^	Yield (%)^a^	ee^b^

1	Ph	H	96	4
2	Ph	Ph	92	22
3	Ph	β-Np	91	20
4	Ph	4-*t-*Bu-C_6_H_4_	83	6
5	Ph	3,5-F_2_-4-OMe-C_6_H_2_	92	26
6	Ph	α-Np	95	24
7	Ph	3-NO_2_-C_6_H_4_	94	32
8	3-NO_2_-C_6_H_4_	4-Br-C_6_H_4_	95	38
9	Cy	3,5-(CF_3_)_2_-C_6_H_3_	91	28
10	Et	3,5-(CF_3_)_2_-C_6_H_3_	94	28
11	3-NO_2_-C_6_H_4_	Ph	92	32
12^c^	3-NO_2_-C_6_H_4_	Ph	95	26
13	3-NO_2_-C_6_H_4_	3,5-F_2_-4-OMe-C_6_H_2_	94	40
14	3-NO_2_-C_6_H_4_	4-*t-*Bu-C_6_H_4_	92	30
15	3-NO_2_-C_6_H_4_	β-Np	95	34
16	3-NO_2_-C_6_H_4_	3,5-(CF_3_)_2_-C_6_H_3_	96	66
**17**	**4-NO_2_-C_6_H_4_**	**3,5-(CF_3_)_2_-C_6_H_3_**	**95**	**70**
18	(*R*)-PhCHCH_3_	3,5-(CF_3_)_2_-C_6_H_3_	94	30
19	(*S*)-PhCHCH_3_	3,5-(CF_3_)_2_-C_6_H_3_	96	28
20	2-Cl-5-NO_2_-C_6_H_3_	3,5-(CF_3_)_2_-C_6_H_3_	96	62
21	3,5-(CF_3_)_2_-C_6_H_3_	3,5-(CF_3_)_2_-C_6_H_3_	96	64
22	3,5-(CO_2_Me)_2_-C_6_H_3_	3,5-(CF_3_)_2_-C_6_H_3_	95	55
23	4-CF_3_-C_6_H_3_	3,5-(CF_3_)_2_-C_6_H_3_	96	60

^a^Isolated yields after 15 h reaction time using 5 mol % of the catalysts. ^b^Determined by HPLC on chiral stationary phase. ^c^The thiourea was tested.

As mentioned before, the combination of solid K_2_CO_3_ as the base and DCM as the solvent was identified as the best system for this reaction and other reaction parameters (catalyst and base amounts, temperature, molar concentration) were systematically investigated next. Table 2 summarizes the most relevant results. Base amounts of 0.25–1 equiv (with respect to aldehyde **5**) can be used without affecting the enantioselectivity, but this results in a slightly reduced reaction rate when using less base. By varying the temperature, dilution, and catalyst loading, as shown in Table 2, **7** was finally obtained with 78% ee and in almost quantitative yield with a reasonably short reaction time when using only 2.5 mol % of the catalyst at −10 °C (Table 2, entry 9). Keeping in mind the fact that significantly larger amounts of catalyst and much longer reaction times were required to achieve a somewhat comparable selectivity and yield with organocatalysts found in the literature [21–23], these results encouraged us to use this methodology to access larger quantities of **8** next.

**Table 2:** Optimization of the asymmetric cascade reaction.

Entry	Catalyst **8a** mol %	*T* (°C)	[**5**]^a^	Time (h)	Yield (%)^b^	ee^c^

1	5 mol %	rt	0.033	15	95	70
2	5 mol %	0°C	0.033	15	95	75
3	5 mol %	−10°C	0.033	15	95	76
4	5 mol %	−20°C	0.033	15	95	60
5	5 mol %	0°C	0.067	6	92	73
6	5 mol %	0°C	0.017	15	91	64
7	10 mol %	rt	0.017	15	94	72
8	2.5 mol %	0°C	0.067	6	93	73
**9**	**2.5 mol %**	**−10°C**	**0.067**	**10**	**98**	**78**

^a^Aldehyde molar concentration. ^b^Isolated yields. ^c^Determined by HPLC on chiral stationary phase.

Thus, under the conditions of entry 9 in Table 2, the reaction was successfully scaled up to a practical 2 mmol scale with unchanged efficiency. After crystallization, we were able to obtain reasonable quantities of (*S*)-**7** with 95% ee and an overall yield of 77%. The availability of both the enantiomers of the catalysts is another important advantage because of the possibility to obtain both the enantiomers of chiral bioactive compounds. In this case, we focused on (*R,R*)-**8a**, which afforded the required (*S*)-**7** (the absolute configuration has been previously determined by vibrational circular dichroism) [31].

### Asymmetric synthesis of **9** by decarboxylation of (*S*)-**7**

**Scheme 2:**
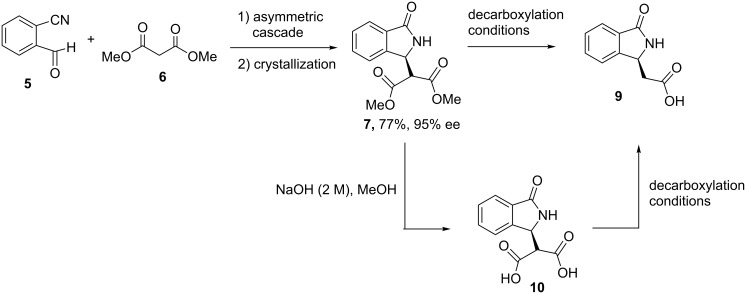
Asymmetric cascade, crystallization and decarboxylation reaction.

Although several methods to obtain *rac*-**9** are available [32–36], it is worth noting that the asymmetric synthesis of **9** is one of the major obstacles when targeting the synthesis of chiral isoindolinones. The recently introduced intramolecular aza-Michael reactions of 2-substituted acrylates gave very poor enantioselectivity (ee <10%) [9], while racemic analogues of **9** were resolved in the past in very low yields [4]. This disappointing picture prompted us to find a viable access route to enantioenriched **9**. We thus investigated the decarboxylation of the chiral dimethyl 2-(*S*)-(1-oxoisoindolin-3-yl)malonate ((*S*)-**7**), according to Scheme 2. We firstly focused on two well-known mild procedures in order to avoid the classical harsh acidic conditions. Disappointingly, modifications of the Krapcho decarboxylation performed with a LiCl/H_2_O/DMF mixture under reflux [36–37] led to partial racemization of the recovered methyl ester of **9** (60% ee), although high yields were observed (Table 3, entry 1). Then, in another attempt, the malonic acid **10** was subjected to the reaction with carbonyldiimidazole (CDI) under different conditions but also in combination with a piperazine. This method has been reported for a number of malonic acids [38] to afford the respective monoacid or directly the mono-amide derivative when the decarboxylation is performed in the presence of amines. However, we were not able to isolate the target compounds since we observed very low conversions and decomposition products. Therefore, we focused on the classical procedures carried out under acidic conditions. Luckily, the decarboxylation of both diester **7** and diacid **10** was particularly encouraging when performed in 6 M HCl solution at reflux in a very short reaction time. This gave the chiral acid **9** in high yield with essentially no loss in ee (Table 3, entries 4 and 8).

**Table 3:** Optimization of the decarboxylation reaction.

Entry	Conditions	Substrate	*T* (°C)	Time	Yield (%)^a^	ee^b^

1^c^	Krapcho decarboxylation	**7**	reflux	2 h	85	60 (37)^d^
2	HCl 6N	**7**	60	96 h	51	36 (60)^d^
3	HCl 6N	**7**	reflux	1 h	96	91 (3)^d^
4	HCl 6N	**7**	reflux	30 min	90	95 (0)^d^
5	CDI/NaOH	**10**	rt	24 h	–	–
6	HCl 3M	**10**	50	24 h	–	–
7	HCl 1M	**10**	80	3 h	46	85 (10)^d^
8	HCl 6M	**10**	reflux	15 min	97	95 (0)^d^

^a^Isolated yield. ^b^Determined by HPLC on chiral column on methyl ester. ^c^The methyl ester of **9** was obtained. ^d^Δee = ee**_5_** − ee**_6_**.

**Scheme 3:**
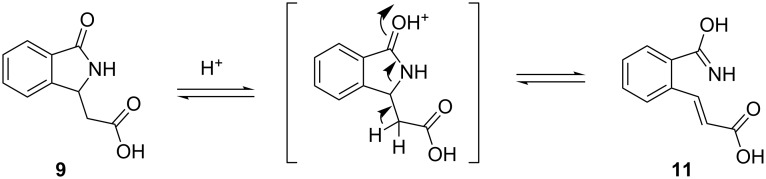
Proposed racemization pathways of isoindolinones **9** via *retro*-Michael process.

On the other hand, partial racemization was detected at longer reaction times (Table 3, entries 2, 3 and 7). This somewhat matched the time-dependent loss in ee observed by Allin et al. in the *N*-deprotection with H_2_SO_4_ of other chiral 3-substituted isoindolinones [39]. The racemization probably occurs on the product **9**, via the mechanism reported in Scheme 3, as a slower process than the decarboxylation itself. The cleavage of the C–N bond and the formation of the acyclic intermediates **11** with the consequent loss of chirality is probably favored by the protonation of the C=O group of the lactam. A similar *retro*-Michael racemization mechanism can be envisioned for the Krapcho decarboxylation, in which the Lewis acid Li^+^ can coordinate the lactam group. In principle, racemization could also occur on **7**. However, under the optimized conditions, the decarboxylation is a faster process and **7** or **10** have never been recovered. Since the decarboxylation does not directly affect the stereocenter, we can confidently assign the absolute configuration of (−)-**9** as (*S*).

### Asymmetric synthesis of bioactive isoindolinones

**Scheme 4:**
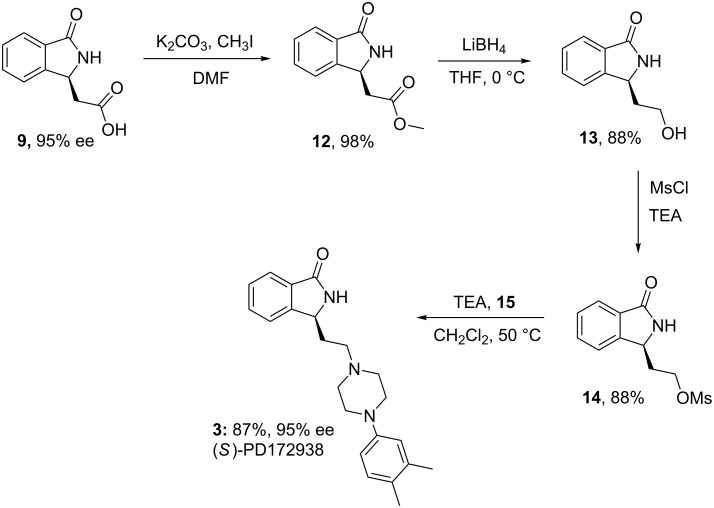
Asymmetric synthesis of (*S*)-PD172938.

Inspired by well-known synthetic routes of racemic derivatives [4–5,8,36], (*S*)-**9** was found to be particularly useful in the asymmetric synthesis of a series of chiral intermediates and bioactive isoindolinones. Firstly, we focused on the reduction of methyl ester **12** to give the chiral alcohol **13**. In this case, LiBH_4_ was particularly effective, leading to **13**in high yield, and most importantly, without a reduction in ee value. To our knowledge, this is the first asymmetric synthesis of **13**. On the other hand, NaBH_4_, previously employed by Belliotti [8] for the reduction of *rac*-**12**, gave less satisfactory results. Then **13** was subjected to reaction with mesyl chloride and the subsequent displacement with 1-(3,4-(dimethylphenyl)pyperazine (**15**) gave **3**, the potent dopamine D_4_ ligand (*S*)-PD172938, in high overall yield (51%), with 95% ee (Scheme 4).

**Scheme 5:**
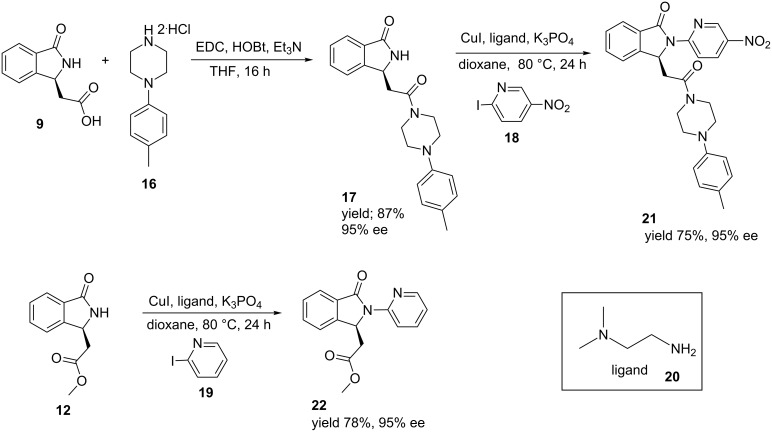
Coupling of chiral acid **9** with *p*-tolylpiperazine and CuI arylation of chiral isoindolinones.

Then, the amide **16**, which is of particular interest in the field of benzodiazepine-receptor agonists, was efficiently obtained by condensation of the chiral acid (*S*)-**9** with the commercially available piperazine **16**. Since most of the bioactive isoindolinones have a heteroaromatic group on the lactam, we focused on the CuI arylation of amides developed by Buchwald [40], previously applied by us to racemic derivatives [36]. Thus, we reacted the two model chiral isoindolinones **12** and **17** (bearing an ester and amide moiety in the side chains, respectively) with two different 2-iodopyridines **18** and **19**. This was done in the presence of CuI and *N*,*N*-dimethylethylenediamine (**20**) as the ligand in dioxane, improving the previous version developed on the racemates (Scheme 5) [36]. This method also allowed us to obtain the analogues **21** and **22** of the bioactive isoindolinones described in Figure 1 in high overall yield (50%) without loss in enantiomeric purity.

## Conclusion

Recently developed (*R,R*)-1,2-cyclohexanediamine-based bifunctional ammonium salts were investigated in the cascade reaction of 2-cyanobenzaldehyde with dimethyl malonate for the synthesis of (*S*)-2-(1-oxoisoindolin-3-yl)malonate. Very high yields and good enantioselectivities were obtained with only 2.5 mol % of the catalyst, improving previous versions performed in the presence of bifunctional organocatalysts and other readily available ammonium salts. Then, decarboxylation of this compound and further transformations allowed the synthesis of the Belliotti (*S*)-PD172938 and of other derivatives with diverse biological activities, in about 50% overall yield with 95% ee. Since the developed routes are particularly convenient in comparison to other syntheses reported in literature, further optimization and the synthesis of other bioactive isoindolinones are ongoing in our laboratory.

## Experimental

**(*S*)-Dimethyl 2-(1-oxoisoindolin-3-yl)malonate** (**7**). A mixture of 2-cyanobenzaldehyde **5** (262 mg, 2 mmol), K_2_CO_3_ (276 mg, 2 mmol) and (*R*,*R*)-catalyst **8a** (27 mg, 0.05 mmol, 2.5 mol %) was dissolved in dichloromethane (30 mL, 0.066 M) and cooled to −10 °C while stirring. Within a period of 2 min, dimethyl malonate **6** (245 μL, 2.4 mmol, 1.2 equiv) was added. After 10 h (reaction monitored by TLC), the reaction mixture was filtered through a plug of Na_2_SO_4_. The solvent was removed under reduced pressure. The crude product was purified by column chromatography (silica gel, heptanes/ethyl acetate 1:1) giving the product as a colorless oil in 98% yield (520 mg, 1.96 mmol) and ee 78%. The enantiomers were separated by HPCL using the following conditions: Chiralcel AD-H, *n*-hexane/iPrOH 70:30, 1.0 mL/min, 10 °C, 12.3 min (minor; *R*-enantiomer), 25.5 min (major; *S*-enantiomer). The product was dissolved in a mixture of dichloromethane (6 mL) and heptanes (4 mL) and after crystallization overnight at −20 °C, the solid was filtered off and the solution containing the enantioenriched compound was evaporated and analyzed by chiral HPLC. The resulting enantioenriched product was obtained as a colourless oil in 77% overall yield (400 mg, 1.51 mmol, 95% ee). The spectroscopic data are in agreement with that reported in literature [21–22]. Chiralpack AD column, hexane/iPrOH 8:2, 0.8 mL/min, λ = 254 nm, *t* = 19.4 min, *t* = 29.3 min.

**(*S*)-2-(1-Oxoisoindolin-3-yl)acetic acid (9) from 7.** A flask containing a solution of isoindolinone **7** (320 mg, 1.20 mmol) and HCl 6 M (2 mL) was immersed in a preheated oil bath and refluxed for 30 min. The mixture was extracted with ethyl acetate (3 × 15 mL). The combined organic layers were dried (MgSO_4_), and the solvent was evaporated to give compound **9**, which was purified with silica gel using ethyl acetate. Compound **9** was obtained as a white solid (208 mg, 1.09 mmol, 90%). Mp 170–171 °C (from ethyl acetate); ESIMS (*m*/*z*): 190.18 (M − H)^−^; [α]_D_^22^ −21 (*c* 1.0, methanol); Anal. calcd for C_10_H_9_NO_3_: C, 62.82; H, 4.74; N, 7.33; found: C, 62.72; H, 4.78; N, 7.01; ^1^H NMR (300 MHz, CD_3_OD) 7.76 (d, *J* = 7.5 Hz, 1H), 7.65–7.53 (m, 2H), 7.51–7.48 (m, 1H), 5.09–4.99 (m, 1H), 2.97–2.89 (m, 1H), 2.68–2.48 (m, 1H); ^13^C NMR (100 MHz, CD_3_OD) δ 172.6, 171.2, 146.8, 131.9, 131.3, 128.1, 122.9, 122.6, 53.4, 38.3. The enantiomeric excess was determined by derivatization of the compound into methyl ester **12** or amide **16**.

## Supporting Information

File 1 Complete experimental details and procedures, spectroscopic data, copies of ^1^H NMR, ^13^C NMR and HPLC traces.
