# Supplementary material for: Bifunctional phase-transfer catalysis in the asymmetric synthesis of biologically active isoindolinones
Source: Beilstein J Org Chem. 2015 Dec 15;11:2591–9. doi: 10.3762/bjoc.11.279 (PMC4685908; doi:10.3762/bjoc.11.279)
Supplement: File 1 — Complete experimental details and procedures, spectroscopic data, copies of 1H NMR, 13C NMR and HPLC traces. [file Beilstein_J_Org_Chem-11-2591-s001.pdf]

Supporting Information

for

Bifunctional phase-transfer catalysis in the asymmetric synthesis of biologically active isoindolinones

Antonia Di Mola[1,2], Maximilian Tiffner[3], Francesco Scorzelli[1], Laura Palombi[1], Rosanna Filosa[4], Paolo De Caprariis[2], Mario Waser[3*#] and Antonio Massa[1a*§]

Address: [1]Dipartimento di Chimica e Biologia "A. Zambelli", Università di Salerno, Via Giovanni Paolo II, 132, 84084-Fisciano, SA, Italy, [2]Dipartimento di Farmacia,Università di Salerno, Via Giovanni Paolo II, 132, 84084-Fisciano, SA, Italy, [3]Institute of Organic Chemistry, Johannes Kepler University Linz Altenbergerstraße 69, 4040 Linz, Austria and [4]Dipartimento di Medicina Sperimentale, Università di Napoli, Napoli, Italy

Email: Mario Waser - mario.waser@jku.at; Antonio Massa - amassa@unisa.it;
[§]Fax: +39-089-969603; Tel: +39-089-969565
[#]Tel: +43 732 2468 8748

*Corresponding author

Complete experimental details and procedures, spectroscopic data, copies of ^{1}H NMR, ^{13}C NMR and HPLC traces

Experimental part S2

^{1}H NMR copies, ^{13}C NMR copies S7

HPLC traces S15

Experimental part

Materials and methods

All reactions were performed using commercially available compounds without further purification. Column chromatographic purification of products was carried out using silica gel 60 (70–230 mesh, Merck). The NMR spectra were recorded on Bruker DRX 400, 300, 250 spectrometers (400 MHz, 300 MHz, 250 MHz, ^{1}H; 100 MHz, 75 MHz, 62.5 MHz ^{13}C). Spectra were referenced to residual $CHCl_3$ (7.26 ppm, ^{1}H, 77.23 ppm, ^{13}C) or other not deuterated residual solvents. Coupling constants *J* are reported in Hz. Yields are given for isolated products showing one spot on a TLC plate and no impurities detectable in the NMR spectrum. HPLC analyses were performed using a Waters instrument on a chiral column. Mass spectral analyses were carried out using an Waters 4 micro quadrupole electrospray spectrometer. Elemental analyses for CHNS-O were performed with a FLASHEA 1112 series-Thermo Scientific apparatus.

Catalyst 8a. Obtained as a colourless oil using the strategy described recently.[1] $[\alpha]_D^{21}$ = + 58 (c = 1.00, $CHCl_3$). ^{1}H NMR (300 MHz, δ, $CDCl_3$, 298 K): 1.32-1.46 (m, 1H), 1.52-2.10 (m, 5H), 2.15-2.28 (m, 1H), 2.55-2.67 (m, 1H), 3.15 (s, 3H), 3.25 (s, 3H), 4.31-4.48 (m, 1H), 4.62-4.75 (m, 1H), 5.30 (d, 1H, *J* = 13.0 Hz), 5.38 (d, 1H, *J* = 13.0 Hz), 7.55 (d, 1H, *J* = 9.6 Hz), 7.75 (d, 2H, *J* = 9.2 Hz), 7.98 (s, 1H), 8.01 (s, 2H), 8.16 (d, 2H, *J* = 9.2 Hz), 9.21 (s, 1H) ppm. ^{13}C NMR (75 MHz, δ, $CDCl_3$, 298 K): 24.6, 25.0, 27.3, 35.9, 48.6, 50.6, 50.8, 65.4, 78.2, 117.8, 117.8, 122.5 (q, *J* = 275 Hz), 125.2, 130.0, 133.2, 133.3 (q, *J* = 34 Hz), 142.6, 145.3, 154.8 ppm. ^{19}F NMR (282 MHz, δ, $CDCl_3$, 298 K): -63.0 ppm. IR (film): $\tilde{\nu}$ = 3265, 3206, 3150, 3083, 3053, 2940, 2864, 2324, 1696, 1613, 1599, 1553, 1505, 1373, 1329, 1279, 1175, 1134, 1109, 921, 850, 752, 710, 682, 495, 411 cm^{-1}. HRMS (ESI): *m/z* calcd for $C_{24}H_{27}F_6N_4O_3^+$: 533.1982 [M^+]; found: 533.1993

(*S*)-Dimethyl 2-(1-oxoisoindolin-3-yl)malonate (**7**). A mixture of 2-cyanobenzaldehyde **5** (262 mg, 2 mmol), K_2CO_3 (276 mg, 2 mmol) and (*R*,*R*)-catalyst **8a** (27 mg, 0.05 mmol, 2.5 mol %) was dissolved in dichloromethane (30 mL, 0.066 M) and cooled to −10 °C with stirring. Within a period of 2 minutes dimethyl malonate **6** (245 □L, 2.4 mmol, 1.2 equiv) was added. After 10 h (reaction monitored by TLC) the reaction mixture was filtrated through a plug of Na_2SO_4. The solvent was removed under reduced pressure. The crude product was purified by column chromatography (silica gel, heptanes/ethyl acetate 1:1) giving the product obtained as a colourless oil in 98% yield (520 mg, 1.96 mmol) and ee 78%. Chiralcel AD-H, *n*-hexane/iPrOH 70:30, 1.0 mL/min, 10 °C, 12.3 min

(minor; *R*-enantiomer), 25.5 min (major; *S*-enantiomer). The product was dissolved in a mixture of dichloromethane (6 mL) and heptanes (4 mL) and after crystallization overnight at −20 °C. The solid was filtered off and the solution containing the enantioriched compound was evaporated and analyzed by chiral HPLC afforded enantio-enriched product as a colourless oil in 77% overall yield (400 mg, 1.51 mmol, ee 95%). Spectroscopic data are in agreement with those reported in literature.[2] Chiralpack AD column, hexane/iPrOH 8:2, 0.8 mL/min, λ = 254 nm, *t* = 19.4 min, *t* = 29.3 min.

(*S*)-2-(1-Oxoisoindolin-3-yl)malonic acid (10). To a solution of isoindolinone **7** (40 mg, 0.15 mmol) in a mixture of dichloromethane/methanol 9:1 (1 mL) was added NaOH 2 M (500 µL) in methanol and stirred overnight. The solvent was removed and ethyl acetate added. The aqueous layer was acidified till pH 2 and extracted four times with diethyl ether. The combined organic layers were dried ($MgSO_4$), and the solvent was evaporated to give the pure compound. White solid (35 mg, 0.149 mmol, 99%). M.p. 176-177 °C (from Et_2O). ESI (m/z): 234.2 $(M-H)^-$. $[\alpha]_D^{22}$= - 6.3 (c 0.1 M in methanol). Anal. calcd for $C_{11}H_9NO_5$. Calcd: C, 56.17; H, 3.86; N, 5.96. Found: C, 56.35; H, 4.04; N, 6.02. ^{1}HNMR (300 MHz, D_2O) δ 7.72 (d, 1H, *J* = 6.87 Hz), 7.63-7.59 (m, 1H), 7.54-7.49 (m, 2H) 5.21 (d, 1H, *J* = 8.55 Hz) 3.68 (d, 1H, *J* = 8.55 Hz). ^{13}CNMR (100 MHz, DMSO-d_6) δ 169.7, 169.2, 168.8, 145.8, 133.0, 131.5, 128.7, 124.0, 123.1, 55.8, 55.1. Enantiomeric excesses were determined by derivatization of the compound into methyl ester **12**.

(*S*)-2-(1-Oxoisoindolin-3-yl)acetic acid (9) from 7. A flask containing a solution of isoindolinone **7** (320 mg, 1.20 mmol) and HCl 6 M (2 mL) was immersed in an oil bath preheated to 150 °C and the solution was refluxed for 30 minutes. The mixture was extracted with ethyl acetate (3 × 15 mL). The combined organic layers were dried ($MgSO_4$), and the solvent was evaporated to give compounds **9** which were purified on silica gel using ethyl acetate. White solid (208 mg, 1.09 mmol, 90%). M.p 170-171 °C (from ethyl acetate). ESI (m/z): 190.2 $(M-H)^-$. $[\alpha]_D^{22}$= -21 (c 1.0 in methanol). Anal. calcd for $C_{10}H_9NO_3$. Calcd: C, 62.82; H, 4.74; N, 7.33. Found: C, 62.72; H, 4.78; N, 7.01. ^{1}HNMR (300 MHz, CD_3OD) 7.76 (d, 1H, *J* = 7. 5 Hz), 7.65-7.53 (m, 2H), 7.51-7.48 (m, 1H), 5.09-4.99 (m, 1H), 2.97-2.89 (m, 1H), 2.68-2.48 (m, 1H).^{13}CNMR (100 MHz, CD_3OD) δ 172.6, 171.2, 146.8, 131.9, 131.3, 128.1, 122.9, 122.6, 53.4, 38.3. Enantiomeric excesses were determined by derivatization of the compound into methyl ester **12** or amide **16**.

(*S*)-2-(1-Oxoisoindolin-3-yl)acetic acid (9) from 10. A flask containing a solution of malonic acid **10** (30 mg, 0.12 mmol) and HCl 6 M (0.5 mL) was immersed in an oil bath preheated to 150 °C and the solution was refluxed for 15 minutes. The mixture was extracted with ethyl acetate (3 × 15 mL). The combined organic layers were dried ($MgSO_4$), and the solvent was evaporated to give compound **9** which were purified on silica gel using ethyl acetate. White solid (23 mg, 0.11 mmol, 97%). M.p. 170-171 °C (from ethyl acetate). ESI (m/z): 190.2 $(M\text{-}H)^-$. $[\alpha]_D^{22}$= -23 (c 1.0 in methanol). Enantiomeric excesses were determined by derivatization of the compound into methyl ester **12**.

(*S*)-Methyl 2-(-oxoisoindolin-3-yl)acetic acid (12). In a round botton flask compound **9** (120 mg, 0.63 mmol, 1 equiv) and K_2CO_3 (1.5 equiv) were dissolved in DMF (3 mL). After 30 minutes iodomethane (2 equiv) were added and the mixture was stirred overnight at room temperature. The solvent was removed under reduced pressure and the residue was purified on silica gel using ethyl acetate/petroleum ether 7:3 to give a white solid. Using acid **9** obtained from **7**, yield 98%: (125 mg, 0.61 mmol), ee 95%; mp 145.6–147.7 °C (from ethyl acetate/petroleum ether); $[\alpha]_D^{22}$= -9.4 (c 1.0 in $CHCl_3$). ESI *(m/z)* = 206.2 $(M+H)^+$. Anal. Calcd for $C_{11}H_{11}NO_3$: C, 64.38; H, 5.40; N, 6.83. Found: C, 64.42; H, 5.30; N, 6.78. Spectroscopic data are in agreement with those reported in literature.[3] Chiralpack IA3 column, hexane–*i*PrOH 8/2, 0.6 mL/min, λ= 254 nm t= 16.1 min and 20.3 min). Using acid **9** (20 mg, 0.104 mmol) obtained from **10**, yield 96%, (20 mg, 0.099 mmol), ee. 95%; mp 145°–147°C (from ethyl acetate/petroleum ether); $[\alpha]_D^{22}$= -9.7 (c 1.0 in $CHCl_3$).

Synthesis of Belliotti (*S*)-PD 172938

(*S*)-3-(2-Hydroxyethyl)isoindolin-1-one (13). A solution of ester **12** (40 mg, 0.2 mmol) in freshly distilled THF (1 mL) was stirred under nitrogen atmosphere. Then a solution of $LiBH_4$ 2 M in THF (150 µL, 0.30 mmol) was added and the mixture was stirred for 2 h, the solvent was removed under reduced pressure. Purification of the residue (ethyl acetate/methanol 95:5) gave the pure compound as an oil. Yellow oil (31 mg, 0.175 mmol, 88%), ee 95%. $[\alpha]_D^{22}$= - 9.6 (c 0.25 in $CHCl_3$). ESI (m/z): 178.2 $(M+H)^+$. ^{1}HNMR (300 MHz, $CDCl_3$) 7.85 (d, 1H, *J* =7. 5 Hz), 7.57-7.43 (m, 3H), 4.75 (d app, 1H, *J* =6.1 Hz), 3.997-3.93 (m, 2H), 2.80 (br s, 1H), 2.28-2.24 (m, 1H), 1.73 (m, 1H).^{13}CNMR (60 MHz, $CDCl_3$) δ 170.8, 147.7, 132.1, 131.9, 128.4, 124.1, 122.6, 61.0, 56.2, 37.2. Anal. calcd for $C_{10}H_{11}NO_2$.. Calcd: C, 67.78; H, 6.26; N, 7.90. Found: C, 67.76; H, 6.36; N, 7.87. Chiralpack IE3 column, hexane–i-PrOH 8/2, 0.6 mL/min, λ= 254 nm t=30.4 min and 34.4 min).

(*S*)-2-(1-Oxoisoindolin-3-yl)ethyl methansulfonate (14). Under nitrogen atmosphere to a solution of alcohol **13** (20 mg, 0.12 mmol) and Et_3N (18 mg, 0.18 mmol, 24 μL) in CH_2Cl_2 was added methansulfonyl chloride (16 mg, 0.14 mmol, 12 μL) and the mixture was stirred at room temperature for 2 h. The solvent was removed under reduced pressure and the residue purified on silica gel (ethyl acetate) to give the pure compound as white solid (26 mg, 0.101 mmol, 88%). M.p. 131-133 °C (from ethyl acetate), ee 95%. $[\alpha]_D^{22}$= - 33.6 (c 0.5 in $CHCl_3$). ESI (m/z): 256.2 $(M+H)^+$. ^{1}HNMR (400 MHz, $CDCl_3$) δ 7.83 (d, 1H, *J* = 7. 3 Hz), 7.63-7.53 (m, 1H), 7.51-7.47 (m, 3H), 4.83 (br s 1H), 4.42-4.41 (m, 2H), 3.03 (s, 3H), 2.51-2.49 (m, 1H), 2.00-1.97 (m 1H). ^{13}CNMR (100 MHz, $CDCl_3$) δ 171.0, 146.4, 132.3, 128.6, 124.0, 122.5, 66.6, 53.6, 37.4, 34.2. Anal. calcd for $C_{11}H_{13}NO_4S$. Calcd: C, 51.75; H, 5.13; N, 5.49.Found: C, 51.68; H, 5.16; N, 5.57.

(*S*)-(2-(4-(3,4-Dimethylphenyl)piperazin-1-yl)ethyl)isoindolin-1-one (*S*)-PD 172938 (3). Under nitrogen atmosphere to a solution of mesylate **14** (20 mg, 0.07 mmol) and Et_3N (18 mg, 0.18 mmol, 24 μL) in CH_2Cl_2 was added 1-(3,4 dimethylphenyl)piperazine (**15**, 16 mg, 0.08 mmol) and the mixture was stirred under reflux overnight. The solvent was removed under reduced pressure and the residue purified on silica gel (ethyl acetate) to give the pure compound as wax like solid (21 mg, 0.06 mmol, 87%), ee 95%. $[a]_D$= - 17 (c 0.75, $CHCl_3$). ESI (m/z): 350.1 $(M+H)^+$. ^{1}HNMR (250 MHz, $CDCl_3$) 7.85 (d, 1H, *J* =7.2 Hz), 7.57-7.42 (m, 4H), 7.02 (d, 1H, *J* = 8.1 Hz), 6.75-6.68 (m, 1H + NH), 4.63 (d app, 1H, *J* = 9.3 Hz), 3.20-3.16 (m, 4H), 2.75-2.54 (m, 6H), 2.23-2.18 (s + m, 7H), 1.80-1.72 (m, 1H).^{13}CNMR (100 MHz, $CDCl_3$) δ 170.7, 170.3, 149.5, 149.4, 147.5, 137.1, 132.0, 131.7, 130.2, 128.3, 123.8, 122.3, 118.3, 114.0, 57.1, 56.5, 53.4, 49.8, 31.3, 20.2, 18.8. Anal. calcd for $C_{22}H_{27}N_3O_2$. Calcd: C, 75.61; H, 7.79; N, 12.02. Found: C, 75.58; H, 7.66; N, 11.97. Chiralpack IA3 column, hexane–i-PrOH 8/2, 0.6 mL/min, λ= 254 nm t=13.2 min and 15.5 min.

Synthesis of benzodiazepine-receptor agonists derivatives

(*S*)-3-{2-[4-(4-Methylphenyl)piperazin-1-yl]-2-oxoethyl}-2,3-dihydro-1*H*-isoindol-1-one (17). Compound **9** (60 mg, 0.31 mmol), 1-(4-methylphenyl)piperazine dihydrochloride (**16**, 53 mg, 0.31 mmol), *N*-(3-dimethylaminopropyl)-*N*-ethylcarbodiimide hydrochloride (58 mg, 0.31 mmol), 1-hydroxybenzotriazole hydrate (41 mg, 0.31 mmol) and triethylamine (100 mg, 1 mmol, 138 μL) in THF (4 mL) were stirred at 25 °C for 16 h, and the reaction mixture then concentrated under reduced pressure. The resulting residue was purified by chromatography on silica gel

($CHCl_3$/methanol 95/5). Yellow oil (96 mg, 0.28 mmol, 87%), ee 95%. $[\alpha]_D^{22}$= -57 (c 1.0 in $CHCl_3$). ESI (*m/z*) 350.2 $(M+H)^+$. ^{1}HNMR (250 MHz, $CDCl_3$) δ 8.06 (d,1H, *J*= 7 Hz) 7.76-7.61 (m, 3H) 7.60 (s, 1H) 7.27(d, 2H, *J*= 8.3 Hz) 7.02 (d, 2H, *J*= 8.4Hz) 5.24 (d, 1H *J*= 10Hz), 4.04-4.00 (m, 2H), 3.76-3.72 (m, 2H), 3.33-3.24 (m, 5H), 2.71-2.60 (m, 1H), 2.46 (s, 3H). ^{13}CNMR (60 MHz, $CDCl_3$) δ 170.3, 168.8, 148.7, 146.6, 132.2, 132.1, 130.7, 130.0, 128.8, 124.3, 122.5, 117.3, 53.6, 50.4, 50.1, 45.5, 41.9, 39.1, 20.6. Anal. Calcd for $C_{21}H_{23}N_3O_2$. Calcd: C, 72.18; H,6.63; N, 12.03. Found: C, 71.99; H, 6.62; N, 11.93. Chiralpack IA3 column, hexane–i-PrOH 8/2, 0.6 mL/min, λ= 254 nm t = 33.4 min, t = 37.5 min).

General procedure for the arylation of isoindolinones

A Schlenk tube was charged with CuI (0.05 mmol) and K_3PO_4 (0.23 mmol), evacuated and backfilled with nitrogen. 2-Iodo-5-nitropyridine or 2-iodopyridine or **18** or **19** (0.17 mmol), *N,N*-dimethylethylenediamine (**20**, 0.07 mmol), and 3-substituted isoindolinones **12** or **17** (0.15 mmol) dissolved in dioxane (1 mL) were added under a nitrogen atmosphere. The reaction mixture was heated at 80 °C for 24 h. The resulting pale-brown suspension was cooled to room temperature. After removing the solvent, the residues were separated by chromatography on silica gel to afford the desired compounds.

(*S*)-2-(5-Nitropyridin-2-yl)-3-(2-oxo-2-(4-*p*-tolylpiperazin-1-yl)ethyl)isoindolin-1-one (21).

Yellow solid (53 mg, 0.112 mmol, 75%). ee 95%. $[\alpha]_D^{22}$= + 45 (c 0.1 in $CHCl_3$). m.p. 188°-189°C (from ethyl acetate /petroleum ether). ESI (*m/z*) = 472.5 $(M+H)^+$. ^{1}HNMR (250 MHz, $CDCl_3$) δ 9.28 (s, 1H), 8.88 (d, 1H, *J*= 9.3 Hz), 8.53 (d, 1H, *J*= 9.3 Hz), 7.77-7.50 (m, 4H), 7.07 (d, 2H, *J*= 8 Hz), 6.81 (d, 2H, *J*= 8 Hz), 6.17 (d, 1H *J*= 9.55 Hz) 3.85-3.84 (m, 2H), 3.55-3.51 (m, 3H), 3.16-3.00 (m, 4H), 2.60-2.49 (m, 1H), 2.26 (s, 3H). ^{13}CNMR (60 MHz, $CDCl_3$) δ 168.3, 168.2, 155.0, 148.8, 146.2, 144.6, 140.2, 134.2, 133.6, 130.6, 130.0, 129.2, 124.8, 124.3, 117.2, 114.3, 57.9, 50.4, 50.2, 45.7, 42.0, 37.5, 20.6. Anal. Calcd for $C_{26}H_{25}N_5O_4$. Calcd: C, 66.23; H, 5.34; N, 14.85. Found: C, 66.11; H, 5.19; N, 14.73. Chiralpack IA3 column, hexane–i-PrOH 8/2, 0.6 mL/min, λ= 254 nm t= 30.02 min, t = 35.8 min).

(*S*)-Methyl 2-[1-oxo-2-(pyridin-2-yl)isoindolin-3-yl]acetate (22). White solid (35 mg, 0.12 mmol, 78%), ee 95%. $[\alpha]_D^{22}$= + 4.6 (c 1.6 in $CHCl_3$).). ESI (m/z): 283.12 $(M+H)^+$. Anal. Calcd for $C_{16}H_{14}N_2O_3$. Calcd: C, 68.07; H, 5.00; N, 9.92. Found: C, 68.38; H, 5.16; N, 9.87. Spectroscopic

data are in agreement with those reported in literature.[4] Chiralpack IA3 column, hexane–*i*PrOH 8/2, 0.6 mL/min, λ= 254 nm t=12.4 min and 13.4 min).

References

[1]Tiffner, M.; Novacek, J.; Busillo, A.; Gratzer, K.; Massa, A.; Waser M. *RSC Adv.* 2015, *5*, 78941.

[2] a) V. More, R. Rohlmann, O. García Mancheño, C. Petronzi, L. Palombi, A. De Rosa, A. Di Mola, A. Massa *RSC Advances* 2012, *2*, 3592. b) S. Tiso, L. Palombi, C. Vignes, A. Di Mola, A. Massa, A. *RSC Adv*. 2013, *3*, 19380.

[3] V. More, A. Di Mola, M. Perillo, P. De Caprariis, R. Filosa, A. Peduto, A. Massa, *Synthesis*, 2011, *18*, 3027

[4] C. Petronzi, S. Collarile, G. Croce, R. Filosa, P. De Caprariis, A. Peduto, L. Palombi, V. Intintoli, A. Di Mola, A. Massa *Eur. J. Org. Chem*, 2012, 5357.

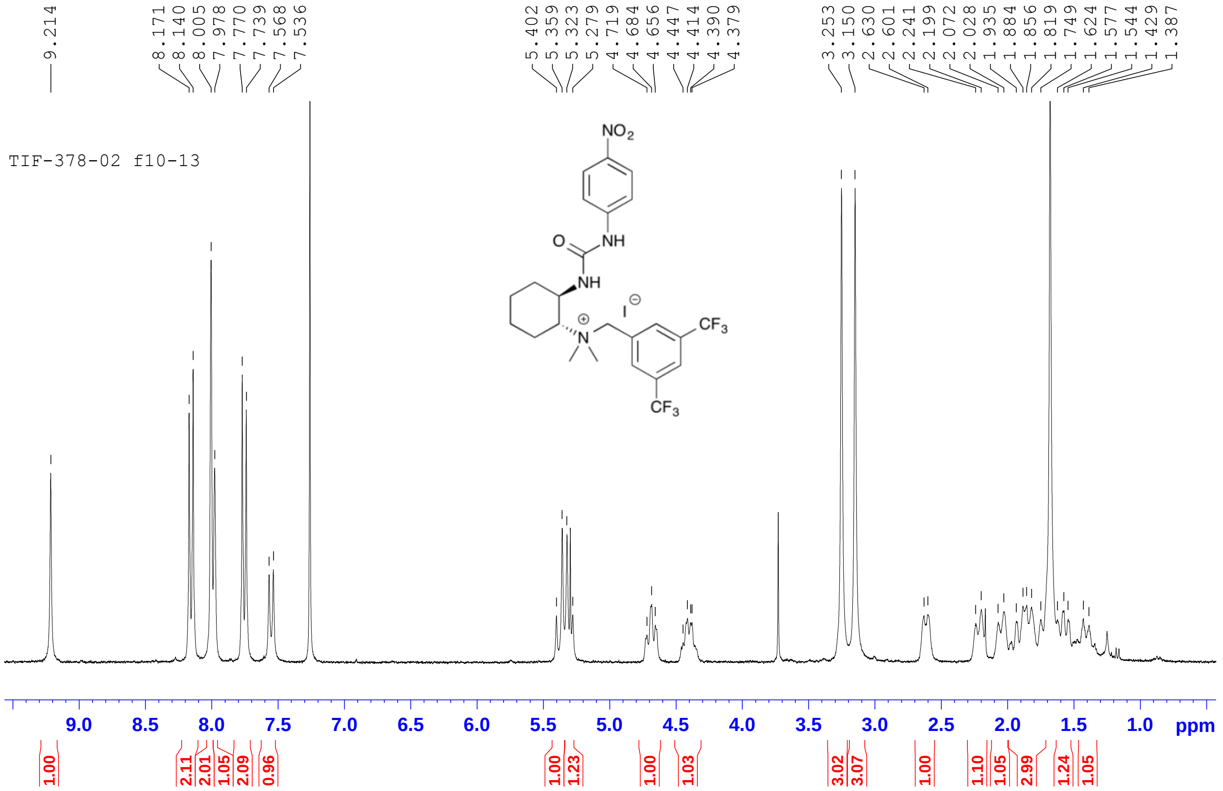
9.214
8.171
8.140
8.005
7.978
7.770
7.739
7.568
7.536
5.402
5.359
5.323
5.279
4.719
4.684
4.656
4.447
4.414
4.390
4.379
3.253
3.150
2.630
2.601
2.241
2.199
2.072
2.028
1.935
1.884
1.856
1.819
1.749
1.624
1.577
1.544
1.429
1.387
TIF-378-02 f10-13
NO2
NH
O
NH
I
N
CF3
CF3
9.0
8.5
8.0
7.5
7.0
6.5
6.0
5.5
5.0
4.5
4.0
3.5
3.0
2.5
2.0
1.5
1.0
ppm
1.00
2.11
2.01
1.05
2.09
0.96
1.00
1.23
1.00
1.03
3.02
3.07
1.00
1.10
1.05
2.99
1.24
1.05

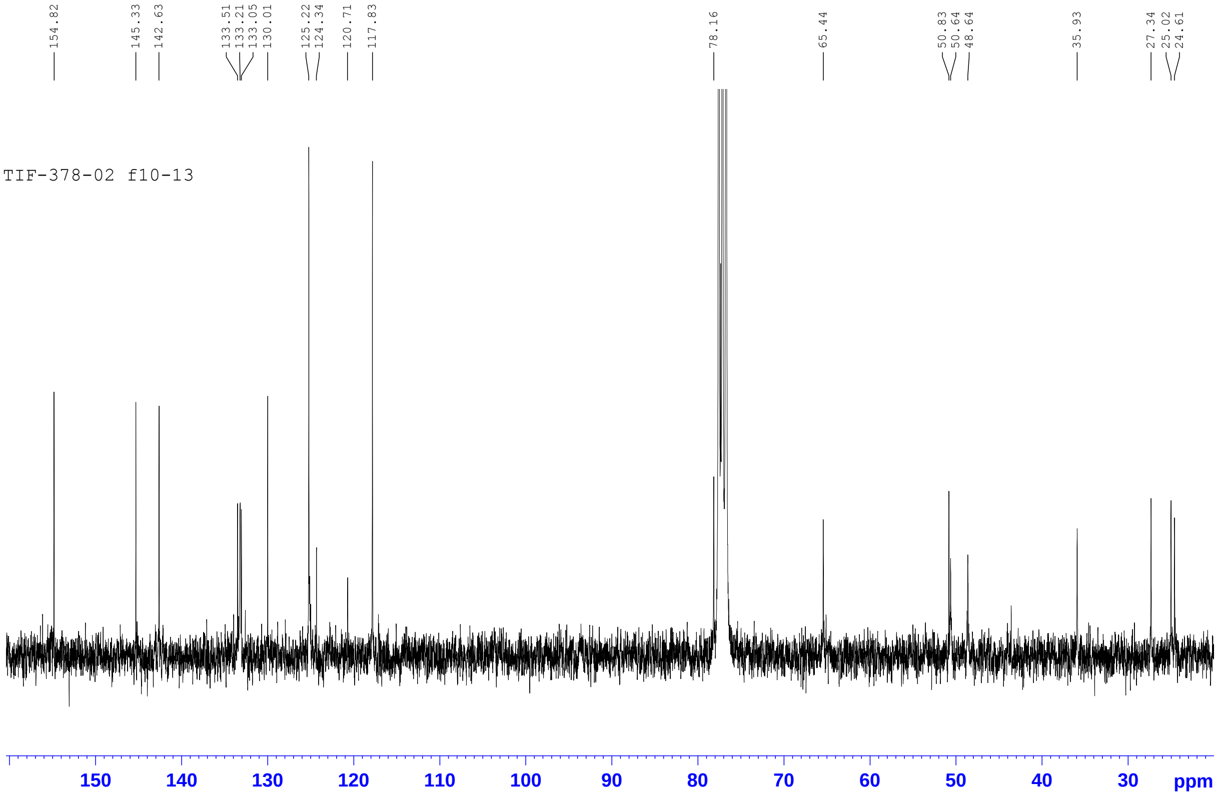
154.82
145.33
142.63
133.51
133.21
133.05
130.01
125.22
124.34
120.71
117.83
78.16
65.44
50.83
50.64
48.64
35.93
27.34
25.02
24.61
TIF-378-02 f10-13
150
140
130
120
110
100
90
80
70
60
50
40
30
ppm

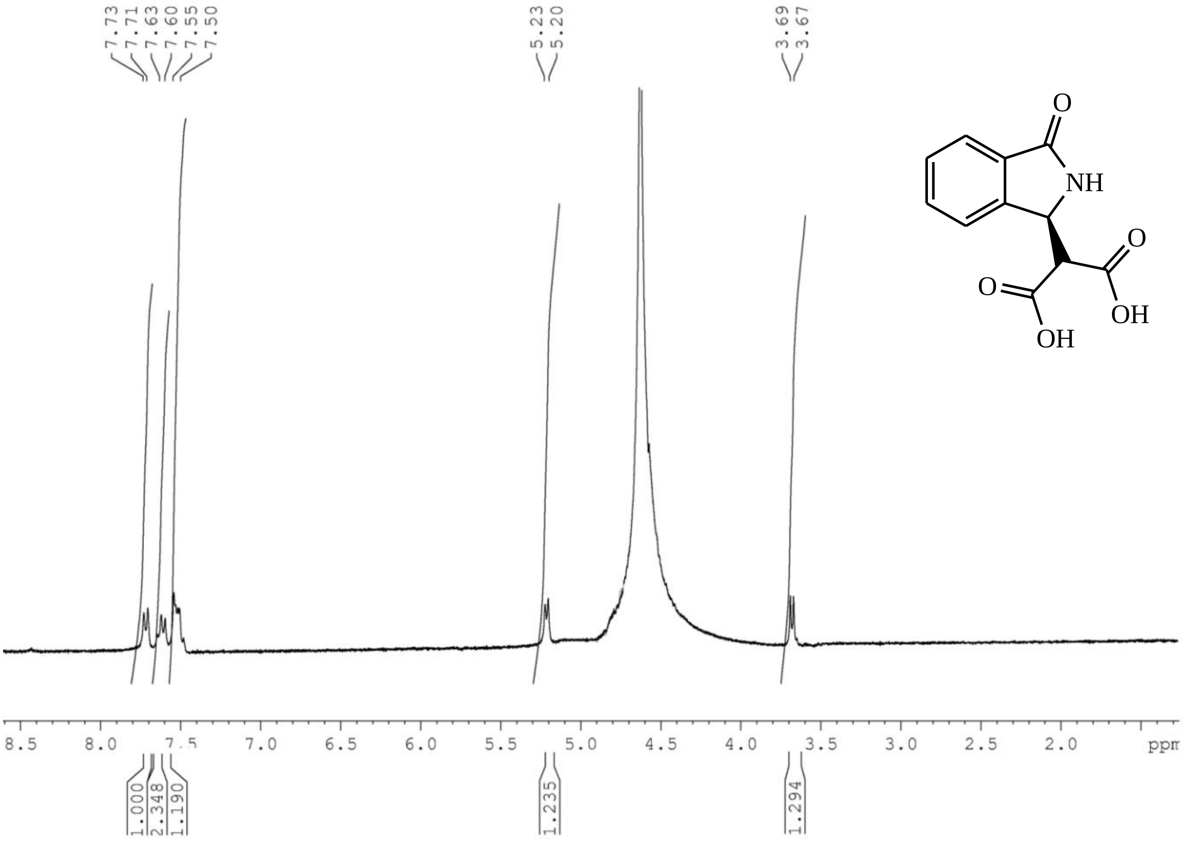
7.73
7.71
7.63
7.60
7.55
7.50
5.23
5.20
3.69
3.67
8.5 8.0 7.5 7.0 6.5 6.0 5.5 5.0 4.5 4.0 3.5 3.0 2.5 2.0 ppm
1.000
2.348
1.190
1.235
1.294
O
NH
O
O
OH
OH

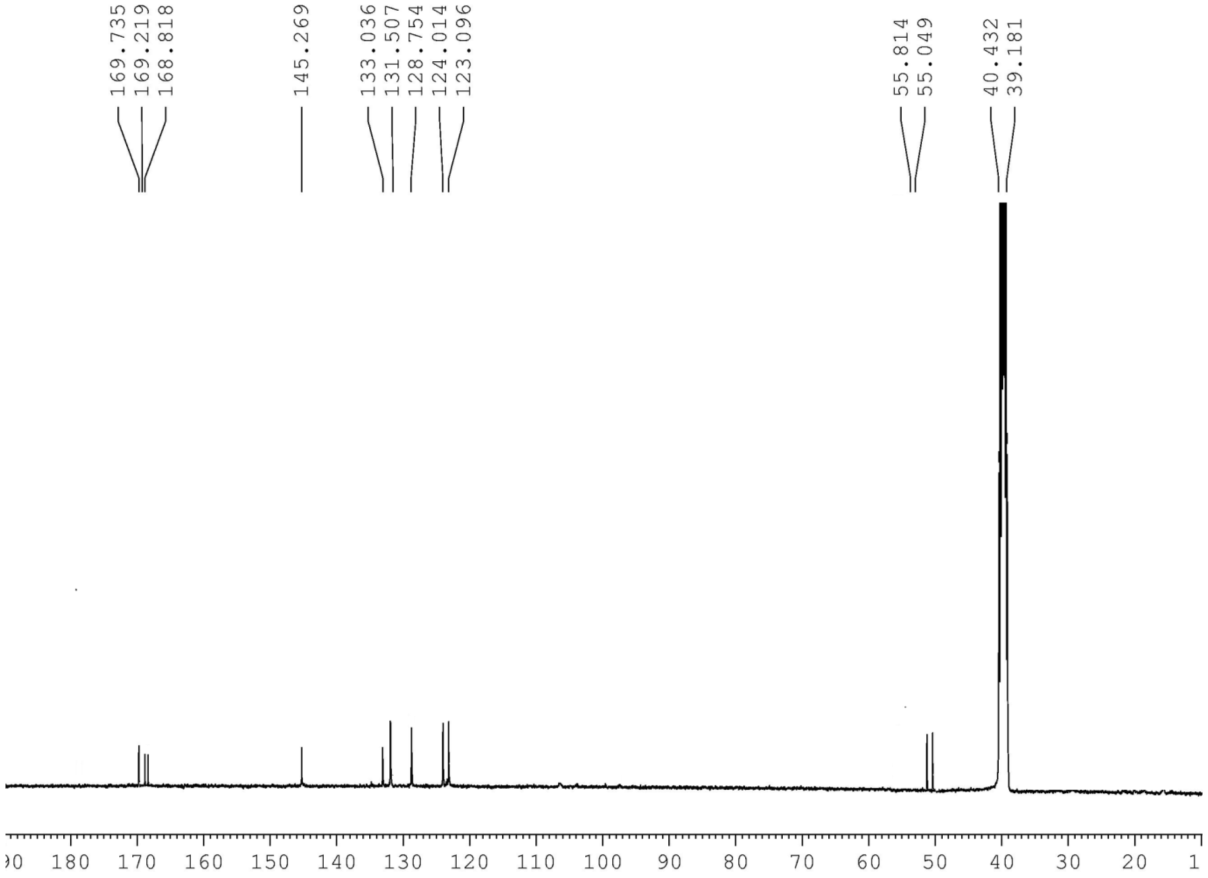
169.735
169.219
168.818
145.269
133.036
131.507
128.754
124.014
123.096
55.814
55.049
40.432
39.181
180 170 160 150 140 130 120 110 100 90 80 70 60 50 40 30 20

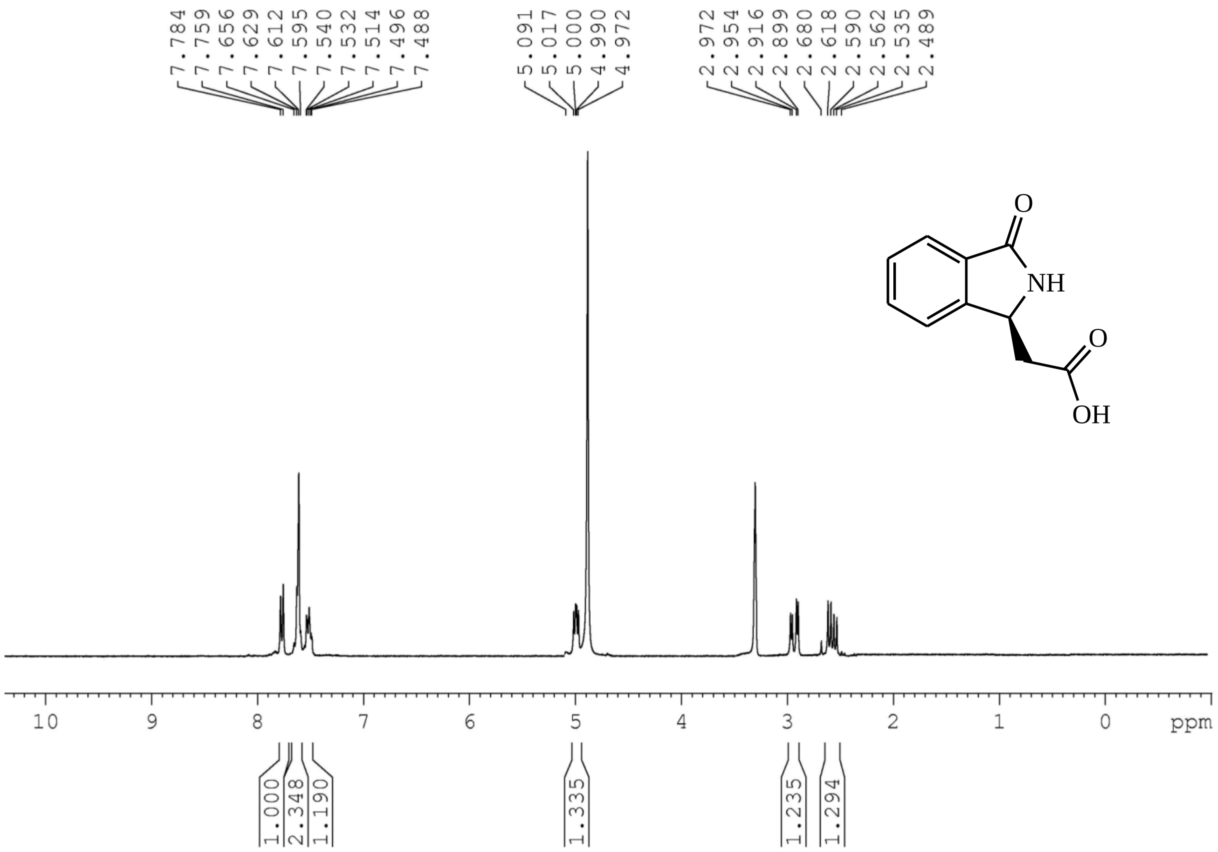
7.784
7.759
7.656
7.629
7.612
7.595
7.540
7.532
7.514
7.496
7.488
5.091
5.017
5.000
4.990
4.972
2.972
2.954
2.916
2.899
2.680
2.618
2.590
2.562
2.535
2.489
O
NH
O
OH
10 9 8 7 6 5 4 3 2 1 0 ppm
1.000
2.348
1.190
1.335
1.235
1.294

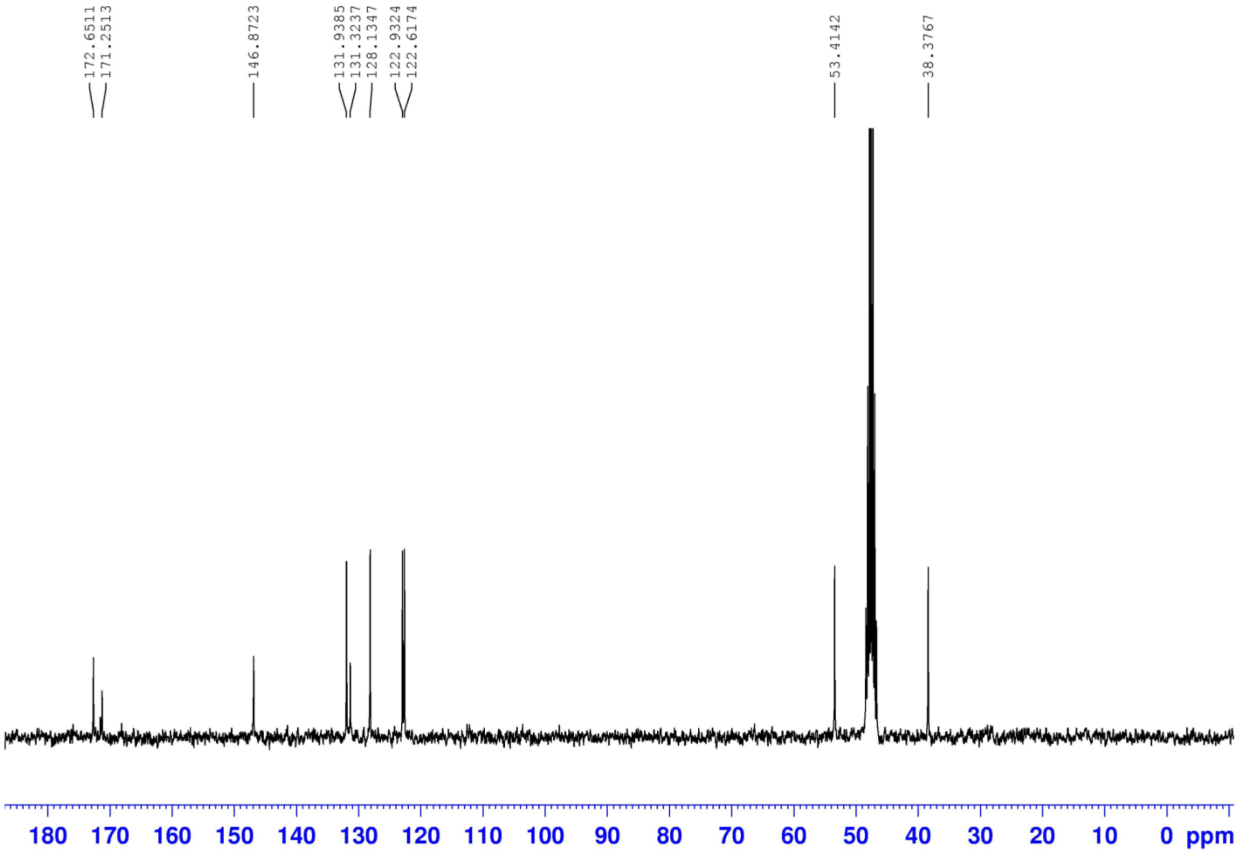
172.6511
171.2513
146.8723
131.9385
131.3237
128.1347
122.9324
122.6174
53.4142
38.3767
180 170 160 150 140 130 120 110 100 90 80 70 60 50 40 30 20 10 0 ppm

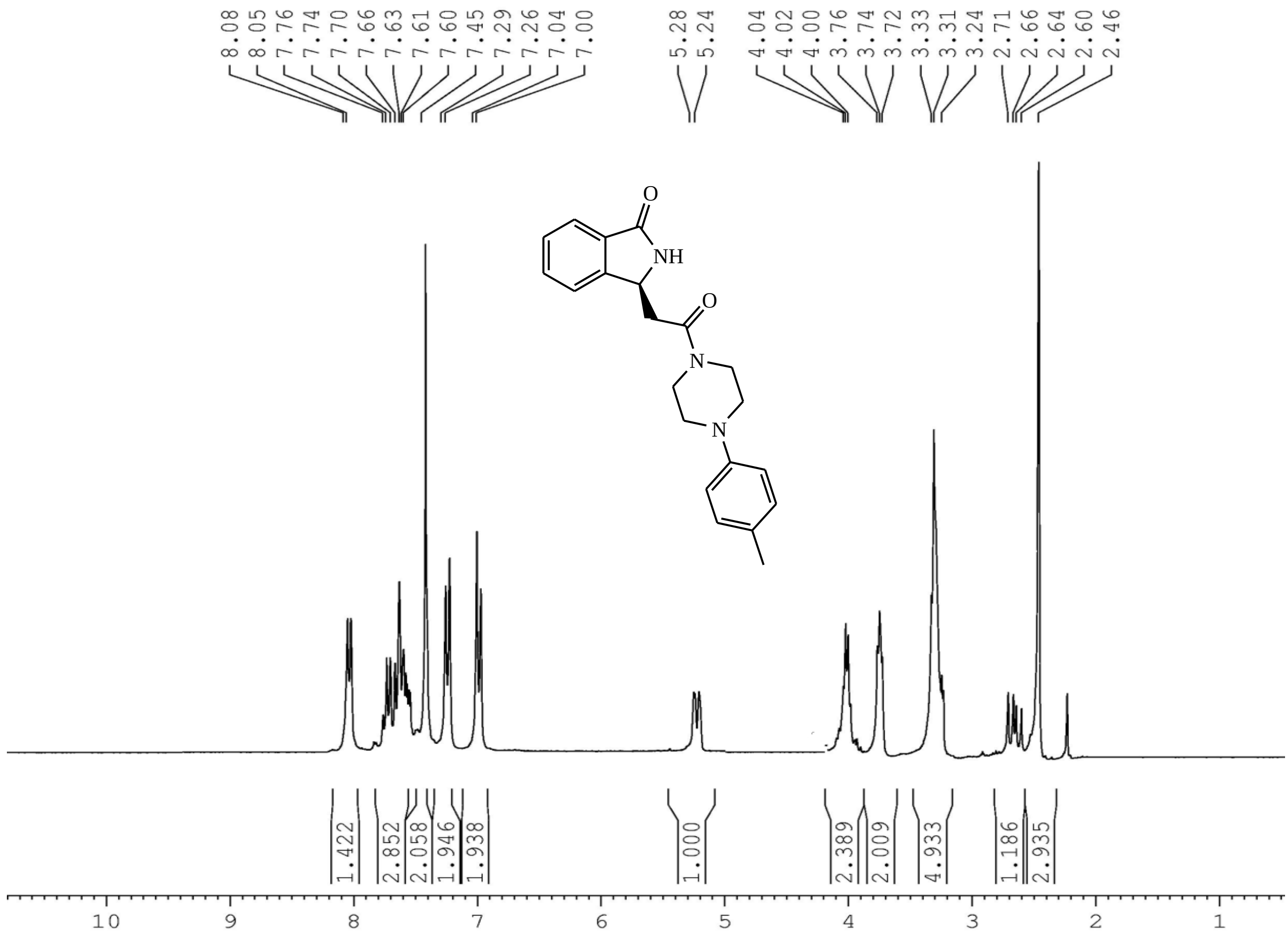
8.08 8.05 7.76 7.74 7.70 7.66 7.63 7.61 7.60 7.45 7.29 7.26 7.04 7.00
5.28 5.24
4.04 4.02 4.00 3.76 3.74 3.72 3.33 3.31 3.24 2.71 2.66 2.64 2.60 2.46
O
NH
O
N
N
1.422 2.852 2.058 1.946 1.938 1.000 2.389 2.009 4.933 1.186 2.935
10 9 8 7 6 5 4 3 2 1

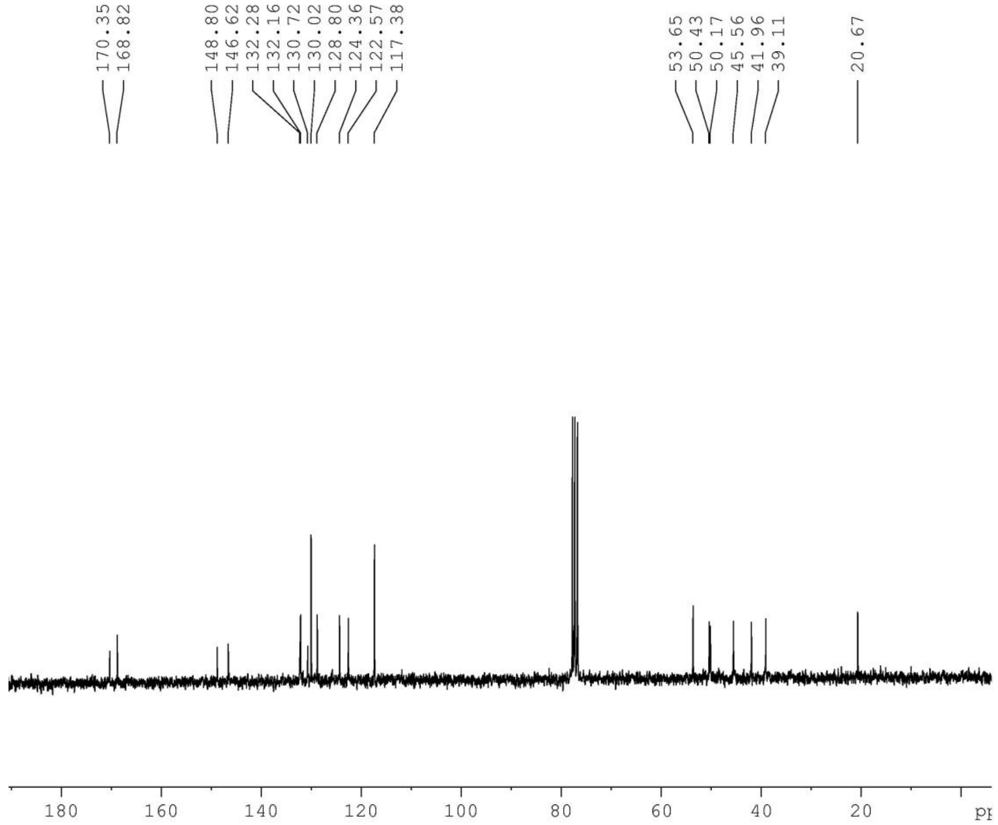
170.35 168.82
148.80 146.62 132.28 132.16 130.72 130.02 128.80 124.36 122.57 117.38
53.65 50.43 50.17 45.56 41.96 39.11
20.67
180 160 140 120 100 80 60 40 20 pp

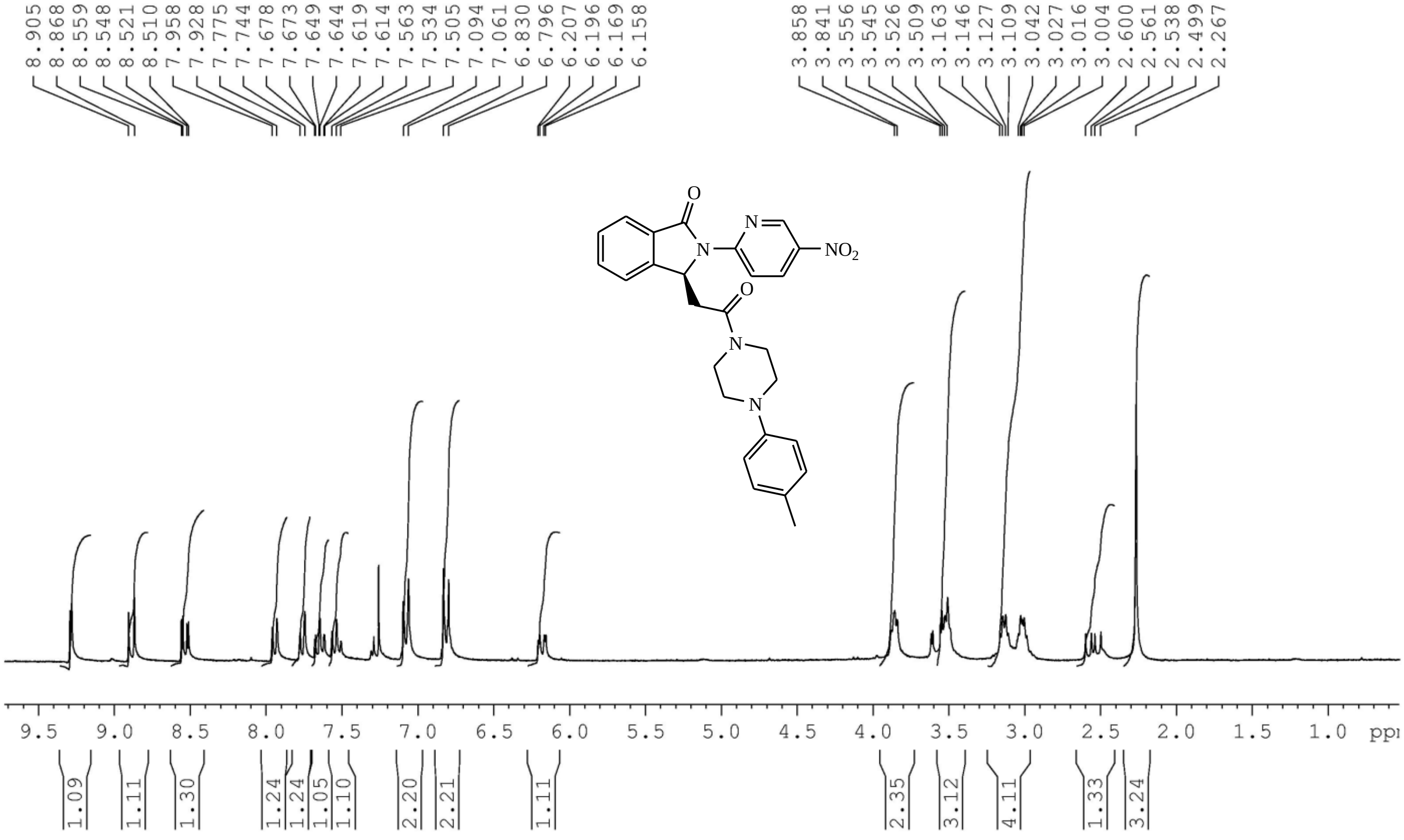
8.905 8.868 8.559 8.548 8.521 8.510 7.958 7.928 7.775 7.744 7.678 7.673 7.649 7.644 7.619 7.614 7.563 7.534 7.505 7.094 7.061 6.830 6.796 6.207 6.196 6.169 6.158
3.858 3.841 3.556 3.545 3.526 3.509 3.163 3.146 3.127 3.109 3.042 3.027 3.016 3.004 2.600 2.561 2.538 2.499 2.267
O
N
N
NO2
O
N
N
9.5 9.0 8.5 8.0 7.5 7.0 6.5 6.0 5.5 5.0 4.5 4.0 3.5 3.0 2.5 2.0 1.5 1.0 ppm
1.09 1.11 1.30 1.24 1.24 1.05 1.10 2.20 2.21 1.11 2.35 3.12 4.11 1.33 3.24

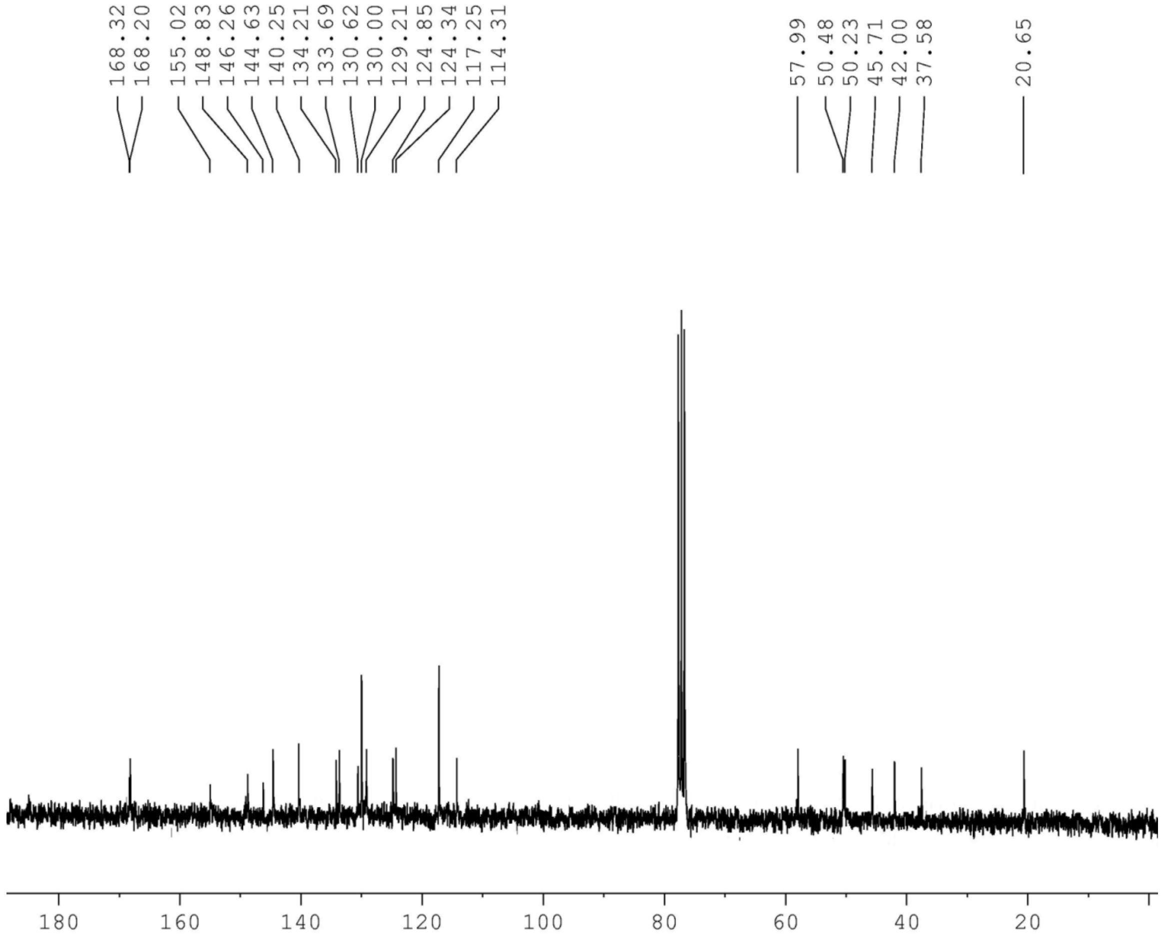
168.32 168.20 155.02 148.83 146.26 144.63 140.25 134.21 133.69 130.62 130.00 129.21 124.85 124.34 117.25 114.31
57.99 50.48 50.23 45.71 42.00 37.58 20.65
180 160 140 120 100 80 60 40 20

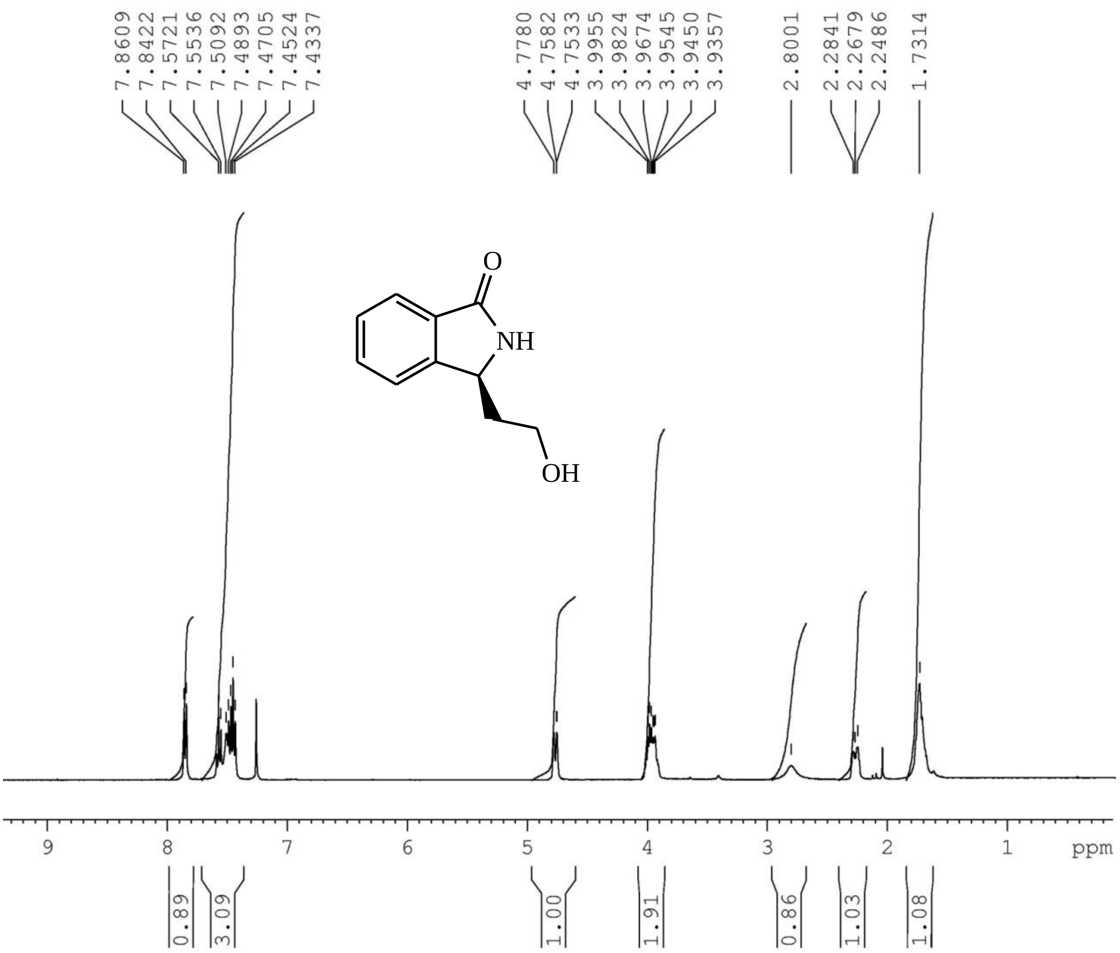
7.8609
7.8422
7.5721
7.5536
7.5092
7.4893
7.4705
7.4524
7.4337
4.7780
4.7582
4.7533
3.9955
3.9824
3.9674
3.9545
3.9450
3.9357
2.8001
2.2841
2.2679
2.2486
1.7314
O
NH
OH
9
8
7
6
5
4
3
2
1
ppm
0.89
3.09
1.00
1.91
0.86
1.03
1.08

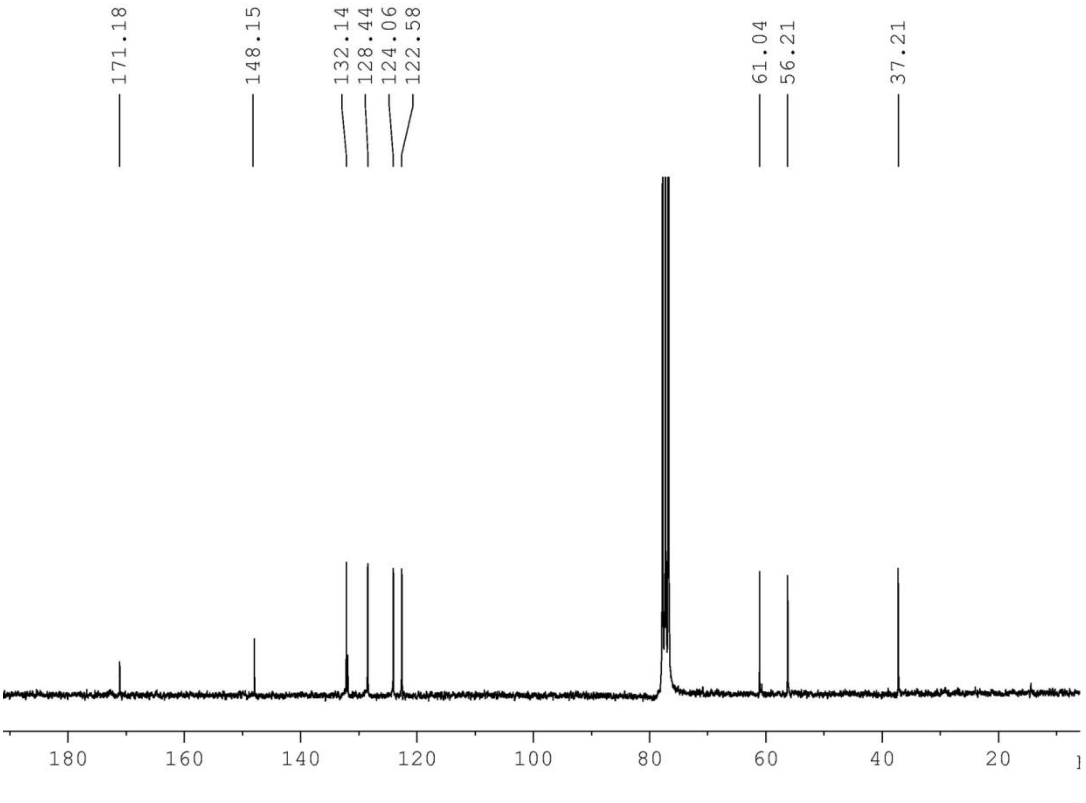
171.18
148.15
132.14
128.44
124.06
122.58
61.04
56.21
37.21
180
160
140
120
100
80
60
40
20

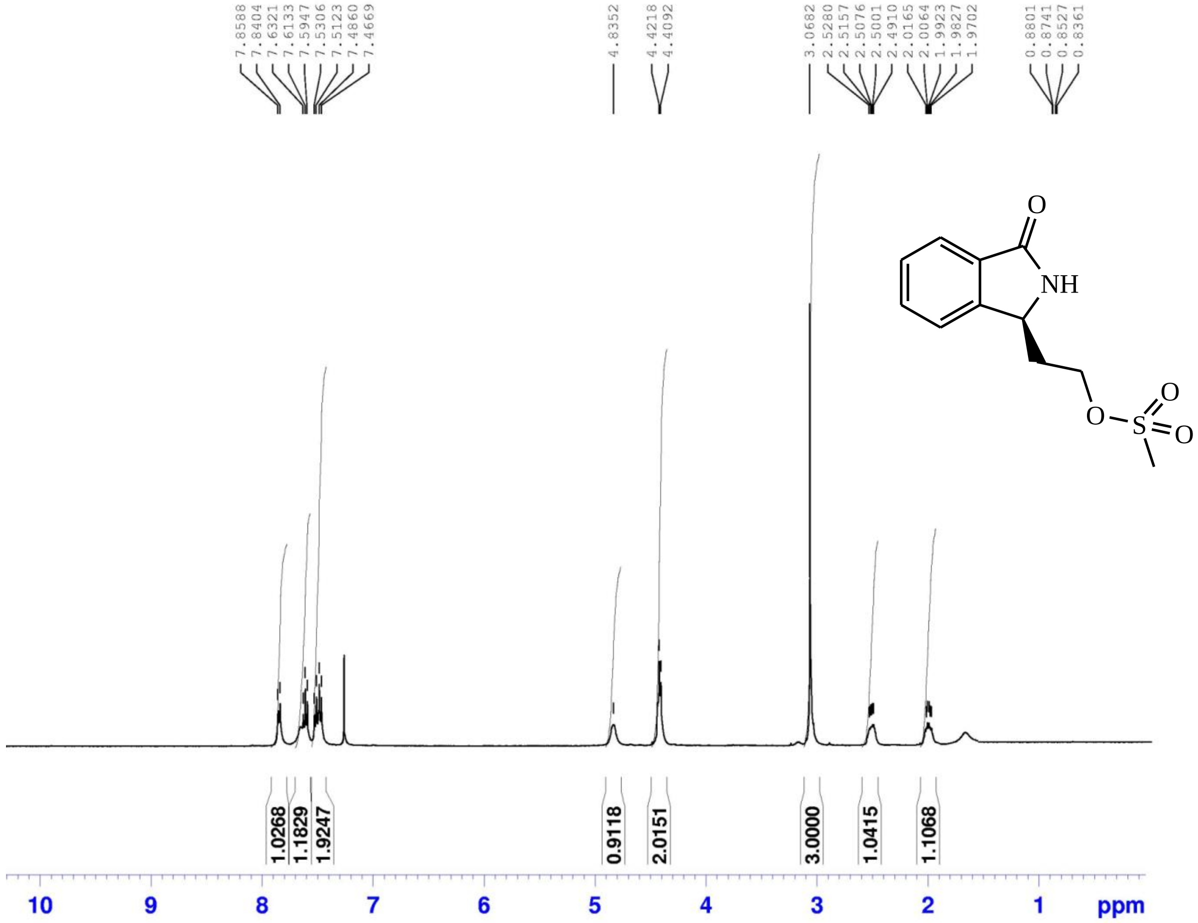
7.8588
7.8404
7.6321
7.6133
7.5947
7.5306
7.5123
7.4860
7.4669
4.8352
4.4218
4.4092
3.0682
2.5280
2.5157
2.5076
2.5001
2.4910
2.0165
2.0064
1.9923
1.9827
1.9702
0.8801
0.8741
0.8527
0.8361
O
NH
O
S
O
O
1.0268
1.1829
1.9247
0.9118
2.0151
3.0000
1.0415
1.1068
10
9
8
7
6
5
4
3
2
1
ppm

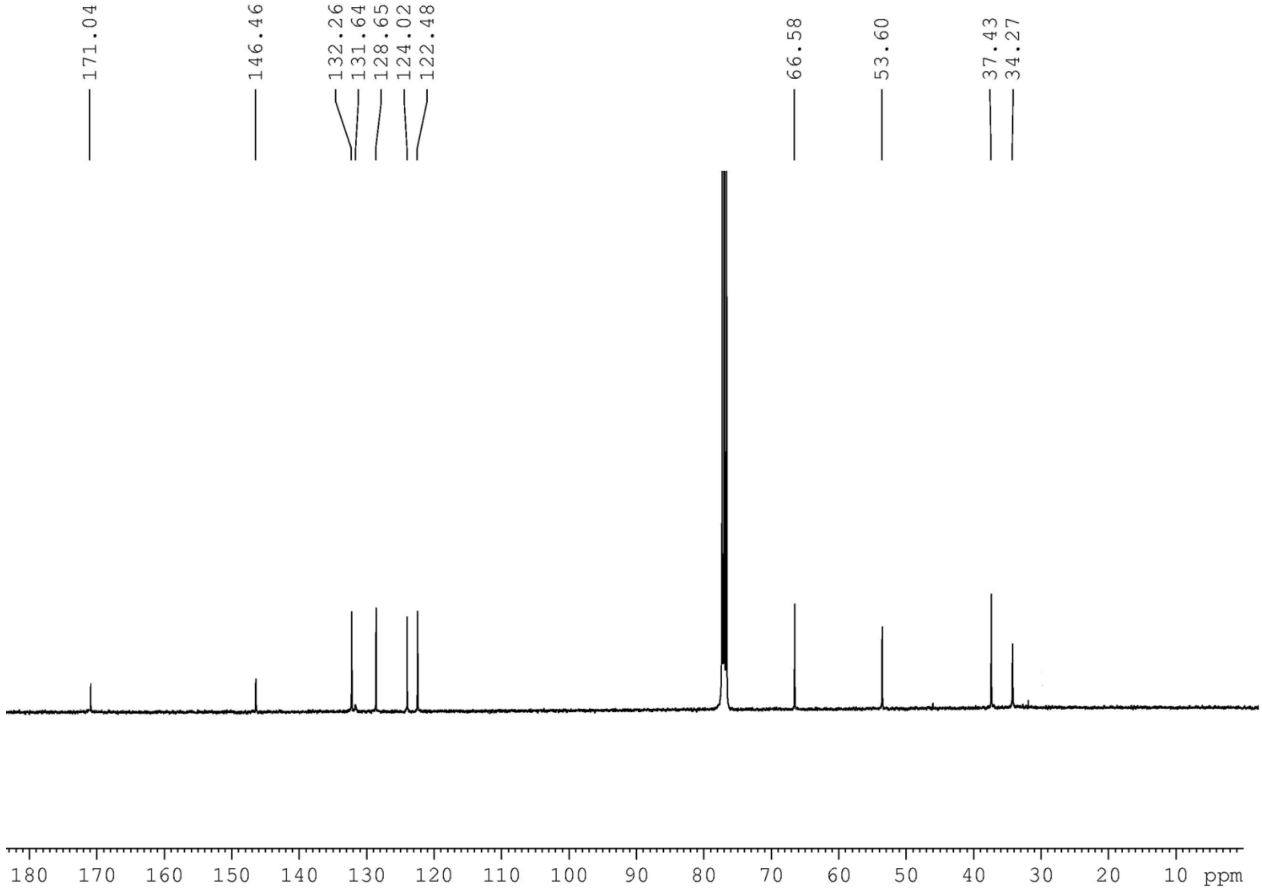
171.04
146.46
132.26
131.64
128.65
124.02
122.48
66.58
53.60
37.43
34.27
180
170
160
150
140
130
120
110
100
90
80
70
60
50
40
30
20
10
ppm

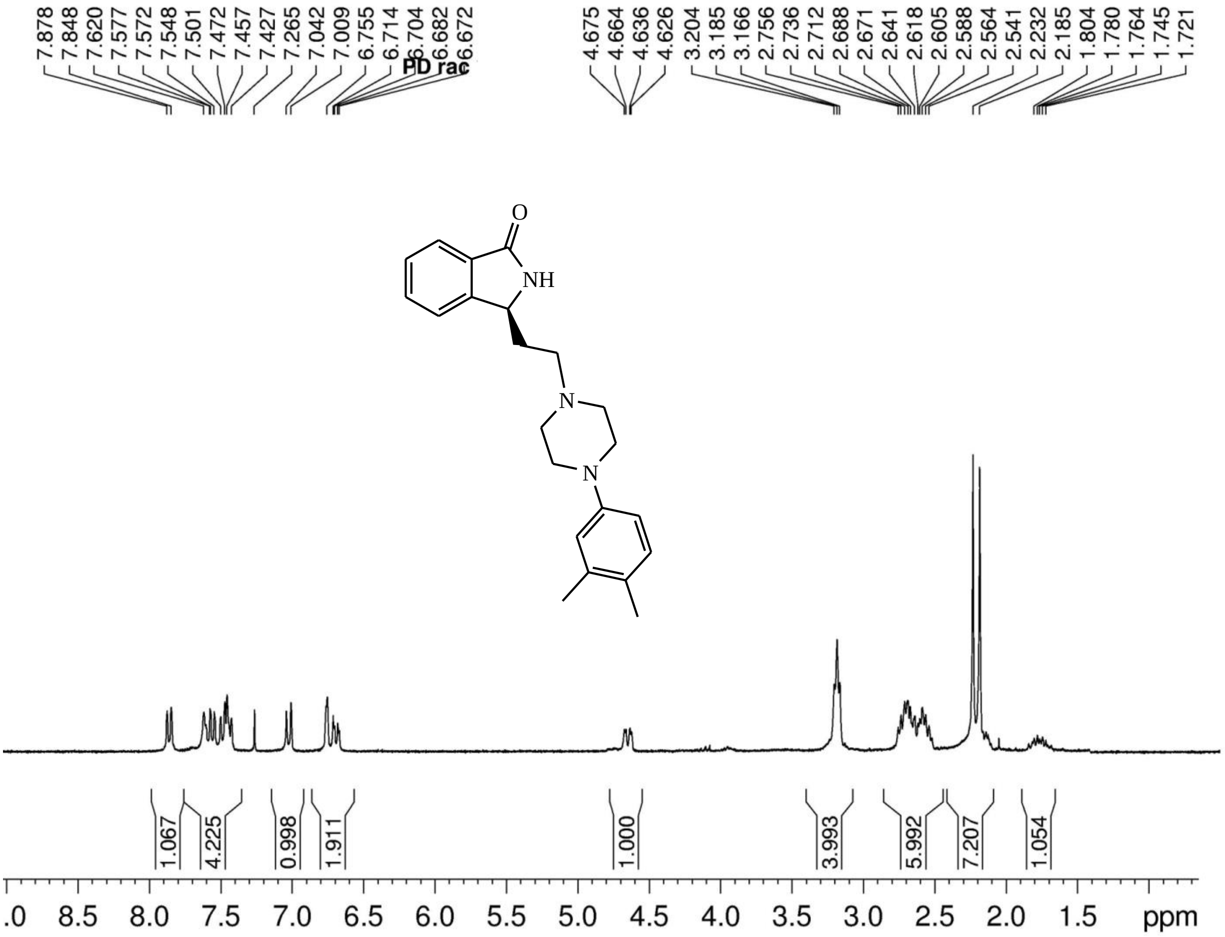
7.878
7.848
7.620
7.577
7.572
7.548
7.501
7.472
7.457
7.427
7.265
7.042
7.009
6.755
6.714
6.704
6.682
6.672
PD rac
4.675
4.664
4.636
4.626
3.204
3.185
3.166
2.756
2.736
2.712
2.688
2.671
2.641
2.618
2.605
2.588
2.564
2.541
2.232
2.185
1.804
1.780
1.764
1.745
1.721
O
NH
N
N
1.067
4.225
0.998
1.911
1.000
3.993
5.992
7.207
1.054
.0 8.5 8.0 7.5 7.0 6.5 6.0 5.5 5.0 4.5 4.0 3.5 3.0 2.5 2.0 1.5 ppm

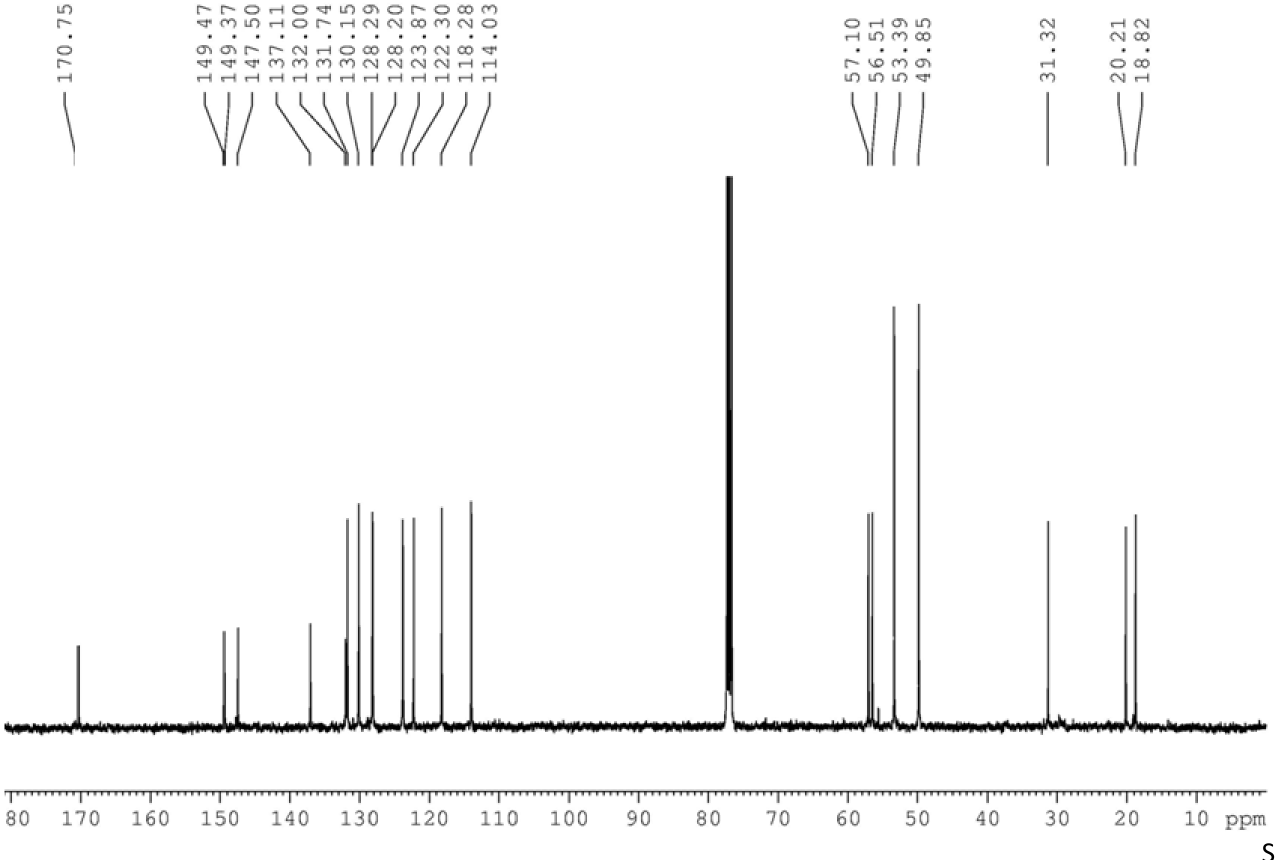
170.75
149.47
149.37
147.50
137.11
132.00
131.74
130.15
128.29
128.20
123.87
122.30
118.28
114.03
57.10
56.51
53.39
49.85
31.32
20.21
18.82
80 170 160 150 140 130 120 110 100 90 80 70 60 50 40 30 20 10 ppm

HPLC traces

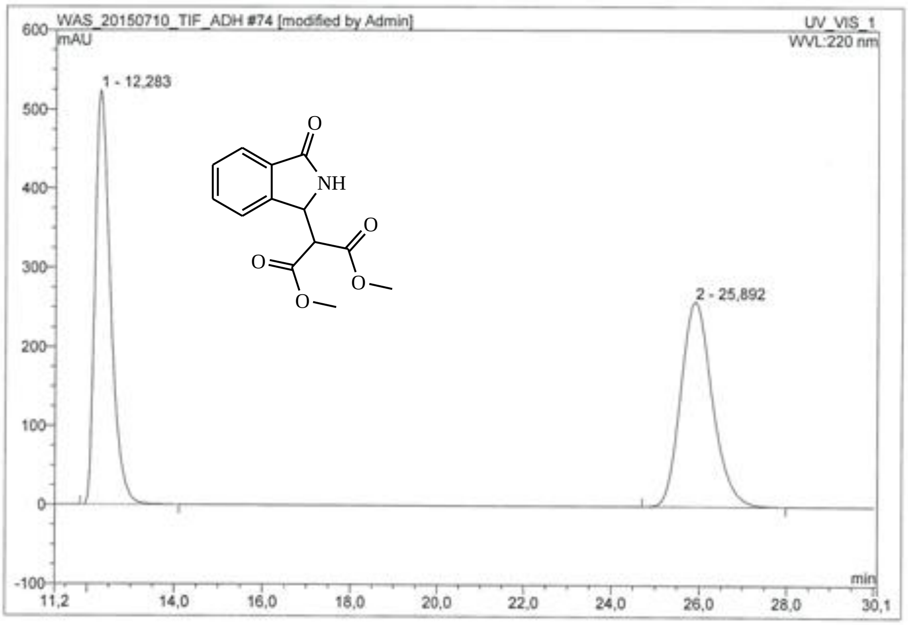

No.	Ret.Time min	Peak Name	Height mAU	Area mAU*min	Rel.Area %	Amount	Type
1	12,28	n.a.	523,708	216,532	49,98	n.a.	BMB
2	25,89	n.a.	258,334	216,724	50,02	n.a.	BMB
Total:			782,041	433,256	100,00	0,000	

Chiralcel AD-H, *n*-hexane: i-PrOH = 70:30, 1.0 mL/min, 10 °C

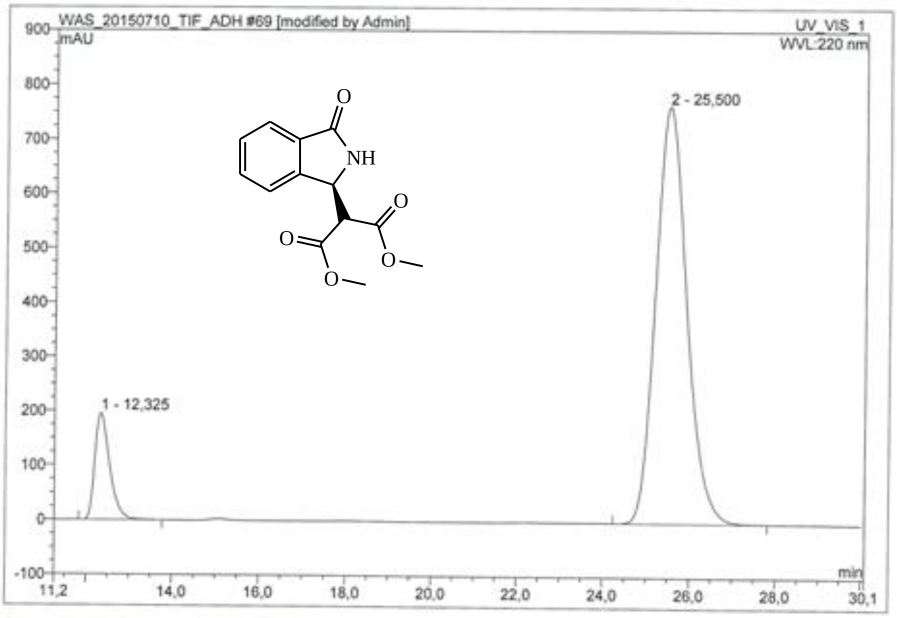

HPLC after reaction

No.	Ret.Time min	Peak Name	Height mAU	Area mAU*min	Rel.Area %	Amount	Type
1	12,33	n.a.	196,037	78,597	11,01	n.a.	BM *
2	25,50	n.a.	764,381	635,070	88,99	n.a.	BMB
Total:			960,417	713,667	100,00	0,000	

Chiralpack AD column, hexane–i-PrOH 8: 2, 0.8 mL/min, λ= 254 nm

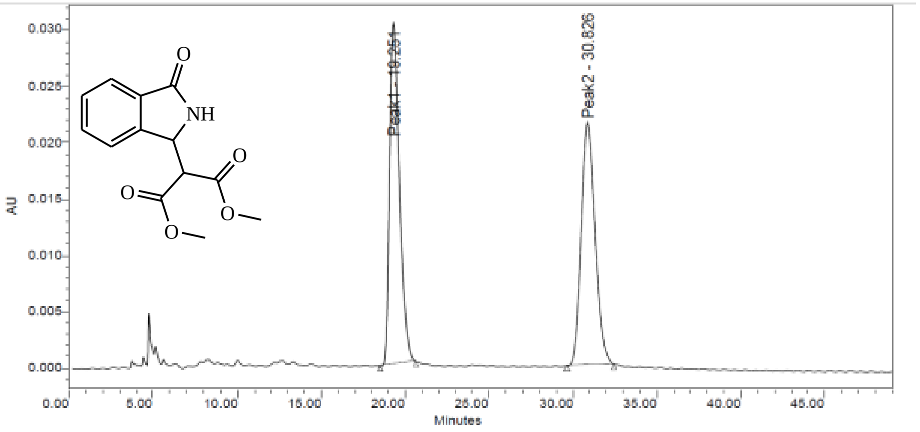

	Peak Name	RT (min)	Area (V*sec)	% Area	Height (V)	% Height
1	Peak1	19.251	1244189	49.96	30389	58.45
2	Peak2	30.826	1246042	50.04	21605	41.55

HPLC after crystallization

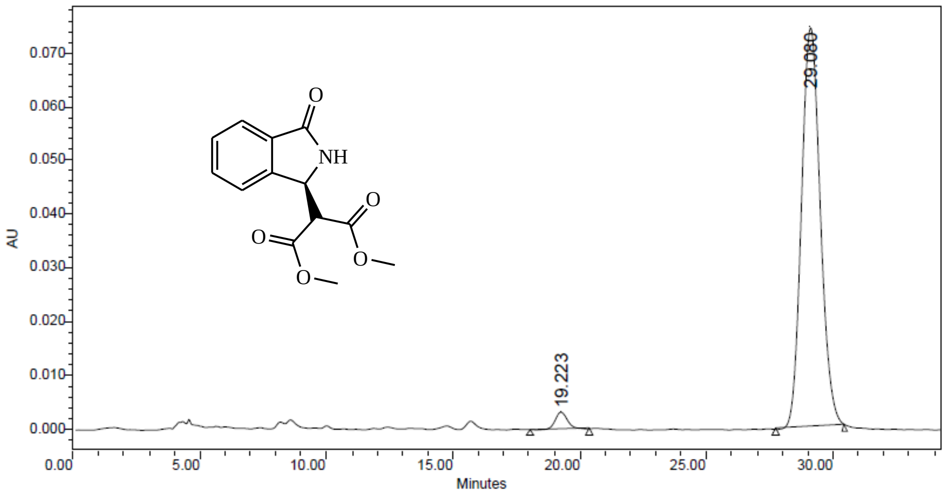

	RT (min)	Area (V*sec)	% Area	Height (V)	% Height
1	19.223	105863	2.68	3151	4.07
2	29.080	3837725	97.32	74247	95.93

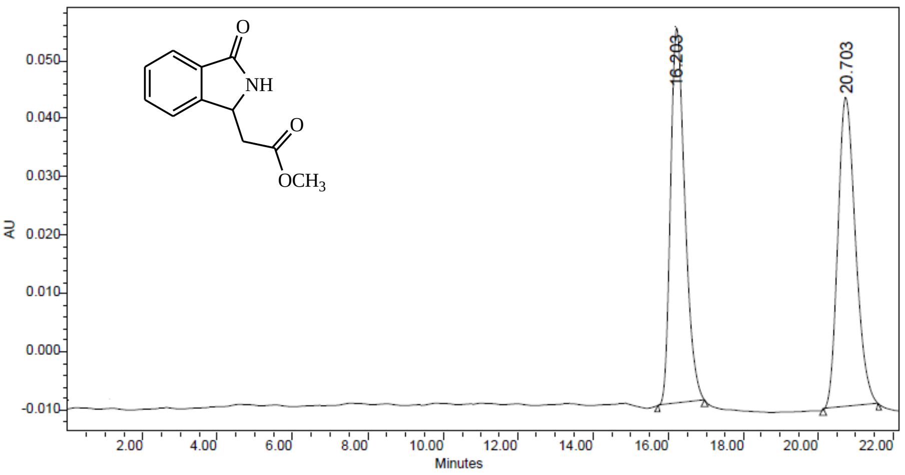

	RT (min)	Area (µV*sec)	% Area	Height (µV)	% Height
1	16.203	1716997	49.65	64395	54.82
2	20.703	1741279	50.35	53067	45.18

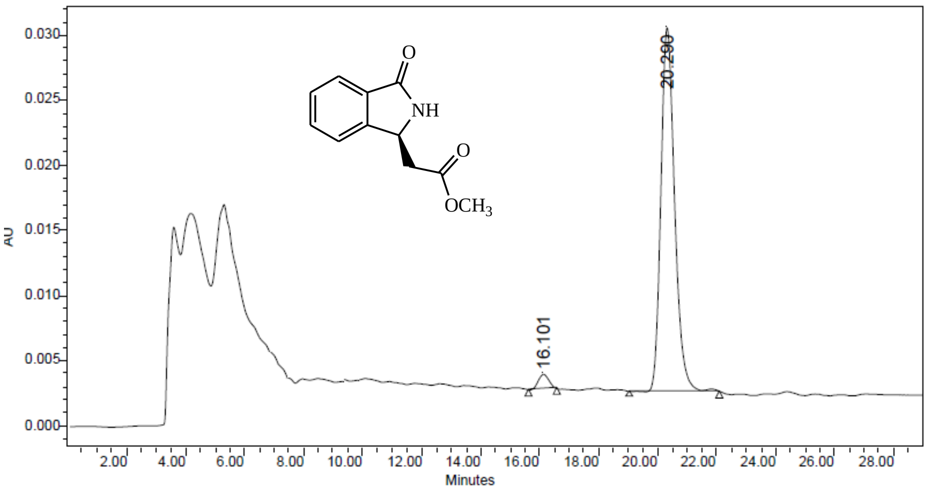

	RT (min)	Area (µV*sec)	% Area	Height (µV)	% Height
1	16.101	245628	2.62	1163	4.00
2	20.290	9120278	97.38	27940	96.00

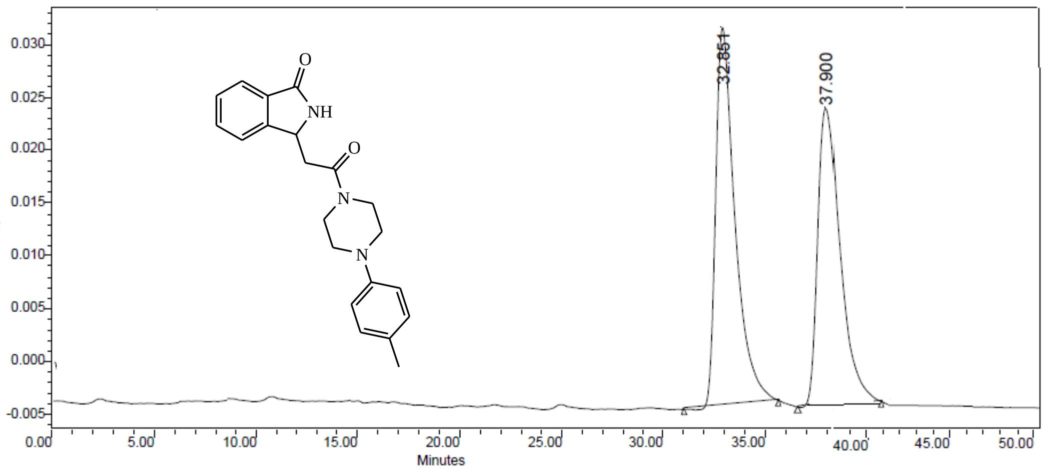

	RT (min)	Area (µV*sec)	% Area	Height (µV)	% Height
1	32.851	2370028	51.54	35757	56.22
2	37.900	2228043	48.46	27840	43.78

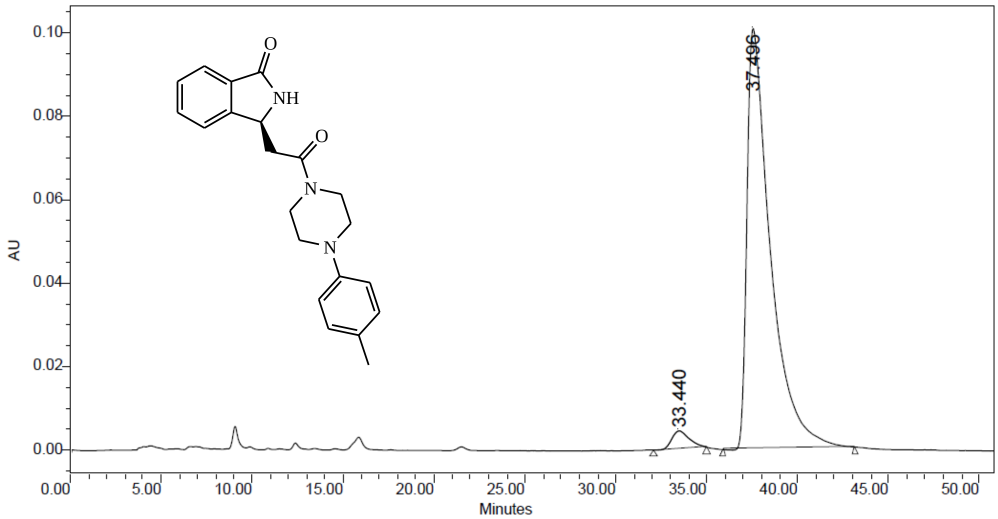

	RT (min)	Area (µV*sec)	% Area	Height (µV)	% Height
1	33.440	252691	2.67	4091	3.89
2	37.496	9202492	97.33	101024	96.11

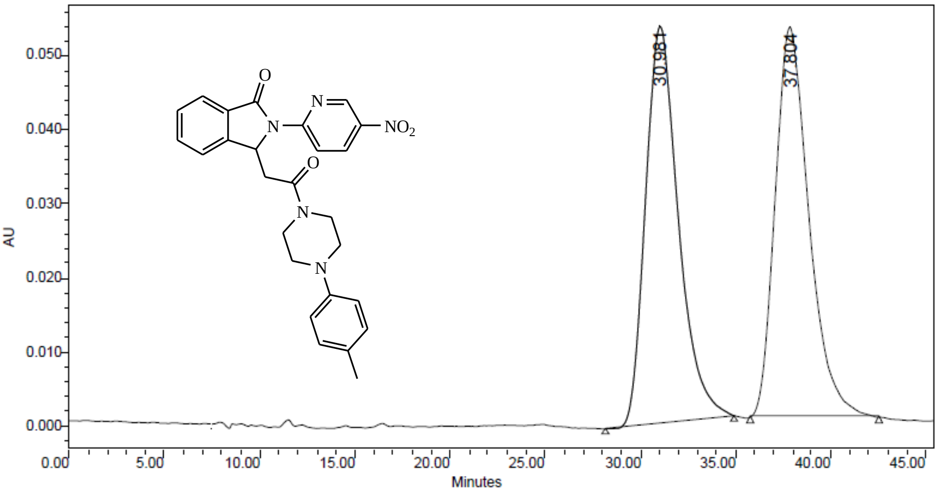

	RT (min)	Area (□V*sec)	% Area	Height (□V)	% Height
1	30.981	6448090	49.73	53696	50.44
2	37.804	6519007	50.27	52751	49.56

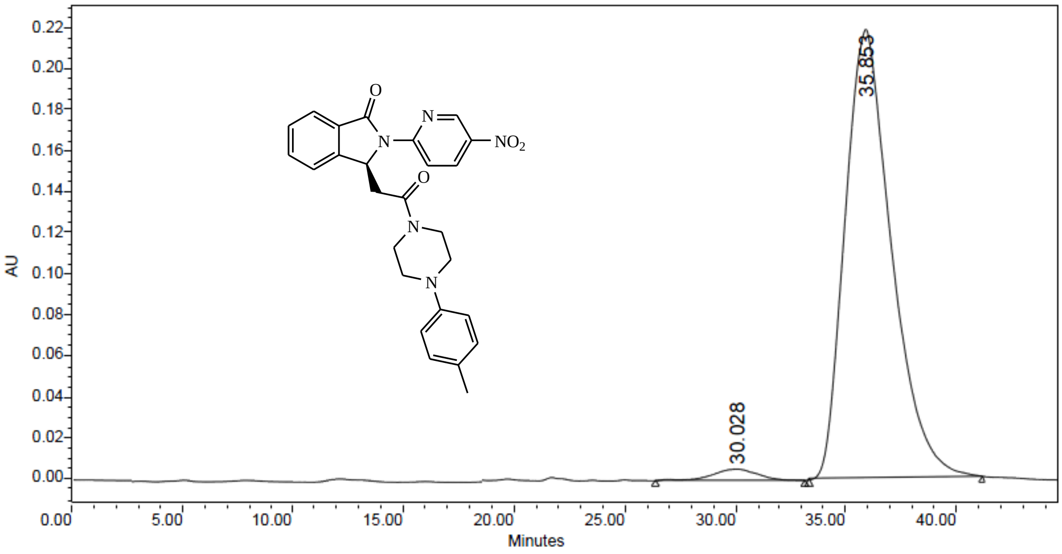

	RT (min)	Area (□V*sec)	% Area	Height (□V)	% Height
1	30.028	791221	2.42	5522	2.46
2	35.853	31964886	97.58	218918	97.54

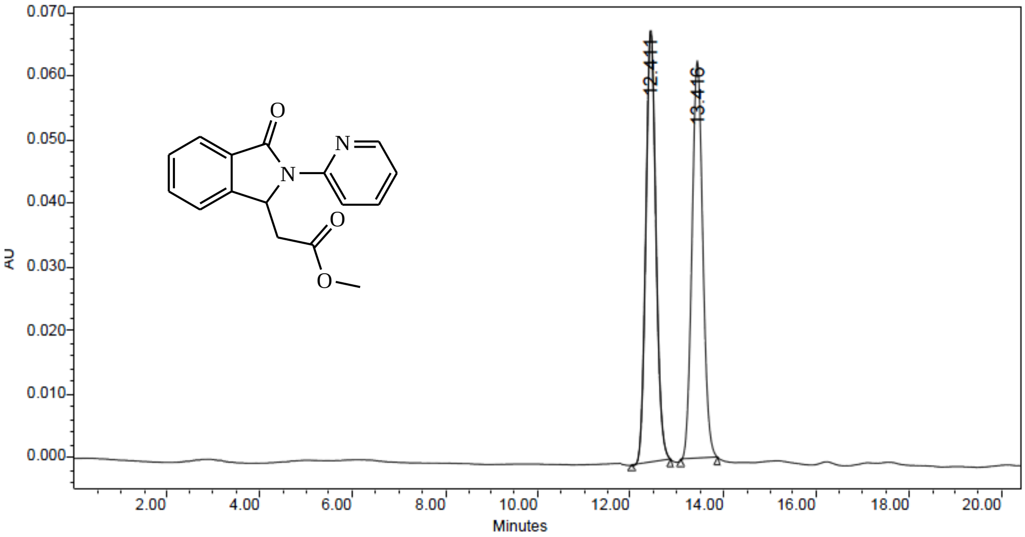

	RT (min)	Area (µV*sec)	% Area	Height (µV)	% Height
1	12.411	1033486	50.42	68160	52.07
2	13.416	1016469	49.58	62730	47.93

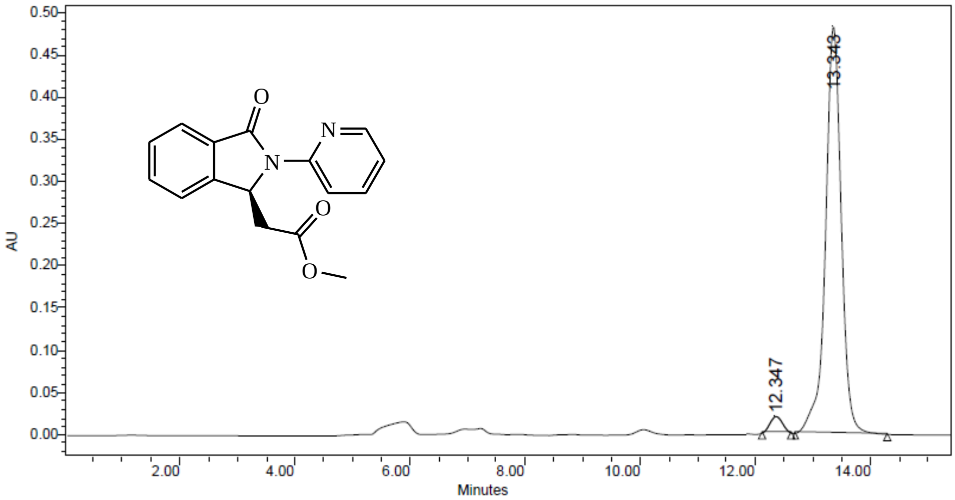

	RT (min)	Area (µV*sec)	% Area	Height (µV)	% Height
1	12.347	68590	2.43	1254	3.08
2	13.343	2754186	97.57	39510	96.92

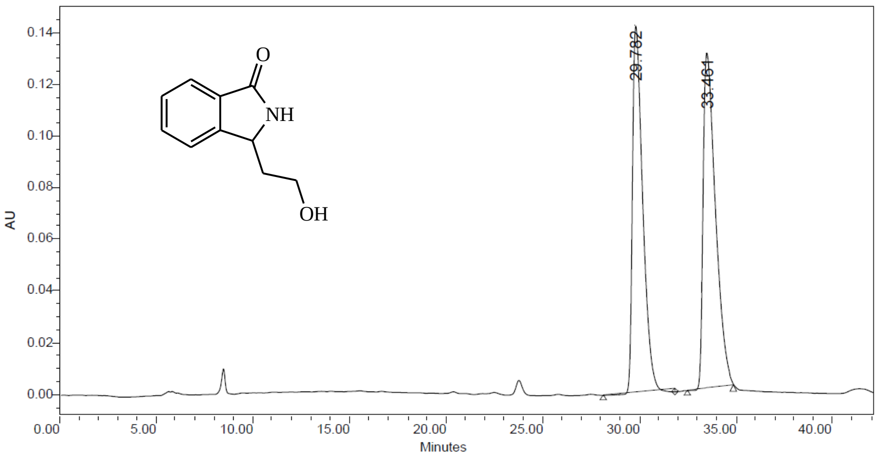

	RT (min)	Area ([illegible]V*sec)	% Area	Height ([illegible]V)	% Height
1	29.782	5248263	48.55	141720	52.37
2	33.461	5562693	51.45	128889	47.63

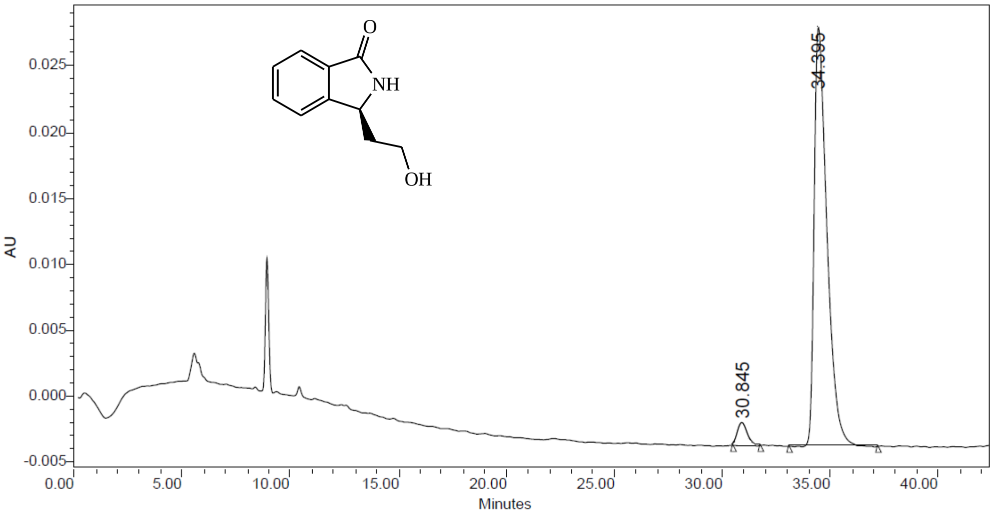

	RT (min)	Area ([illegible]V*sec)	% Area	Height ([illegible]V)	% Height
1	30.845	35317	2.63	1371	4.14
2	34.395	1306972	97.37	31720	95.86

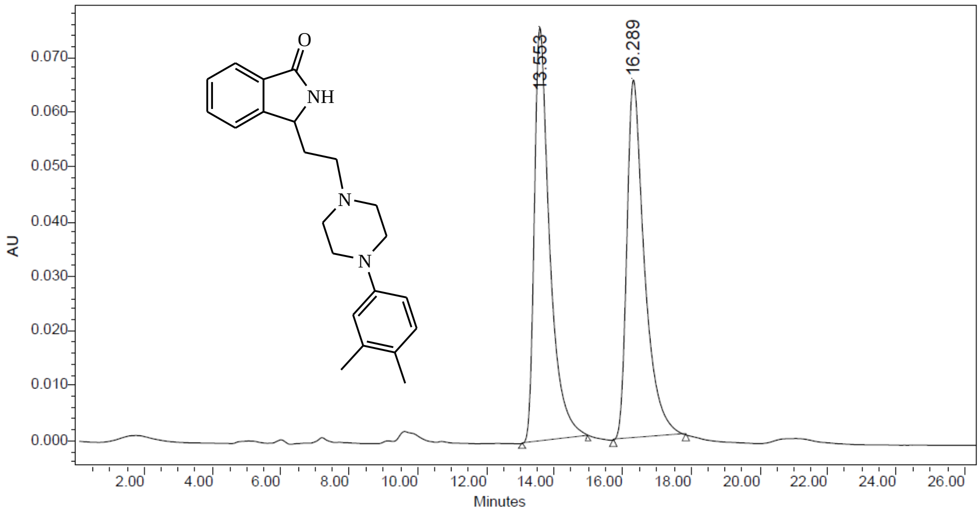

	RT (min)	Area (V*sec)	% Area	Height (V)	% Height
1	13.553	2362551	50.05	75579	53.56
2	16.289	2358183	49.95	65524	46.44

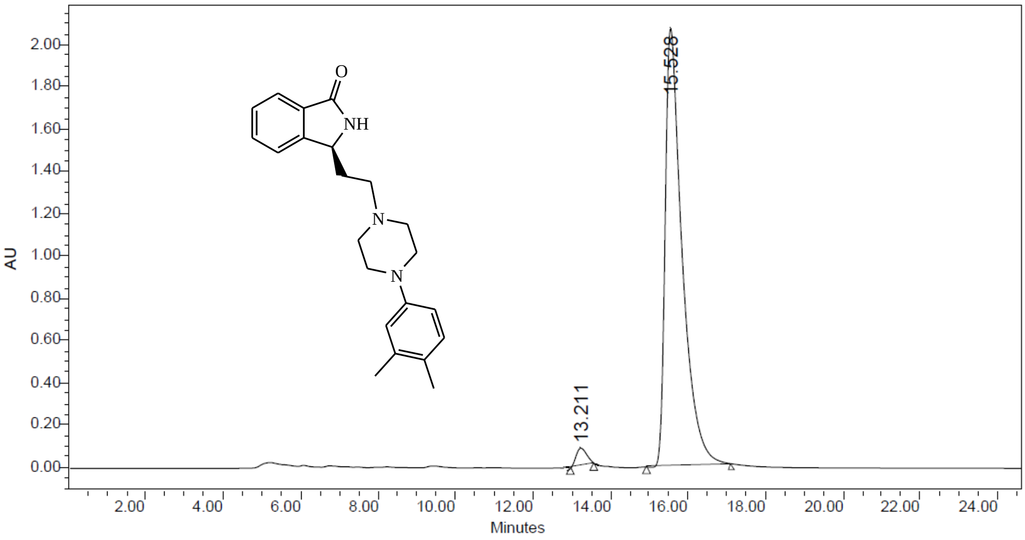

	RT (min)	Area (V*sec)	% Area	Height (V)	% Height
1	13.211	1691049	2.62	86528	4.01
2	15.528	62959216	97.38	2070560	95.99
